# Human simulation model for predicting body temperature, blood pressure, and blood flow rate under various thermal exposures — Part 1: development of physical model for average Japanese male in their 20s

**DOI:** 10.1007/s00484-025-03072-6

**Published:** 2026-02-03

**Authors:** Tomonobu Goto, Zhuoxi Niu, Yuki Chiba, Kentaro Amano, Yoshifumi Saijo

**Affiliations:** 1 Department of Architecture and Building Science, Tohoku University, Aoba 6-6-11-1204, Sendai, 980-8579 Japan; 2 Izumi Consulting Co., Ltd., Tokyo, Japan; 3 Takenaka Research and Development Institute, Inzai, Japan; 4 Department of Biomedical Engineering, Tohoku University, Sendai, Japan

**Keywords:** Human simulation model, Thermal environment, Health risk, Body temperature, Blood flow rate, Blood pressure

## Abstract

In the mechanism of thermal health problems, not only body temperature but also blood pressure and blood flow rate are key factors. Human body simulation is a useful tool for predicting these physiological quantities under various conditions. Therefore, we aimed to develop a new human-body simulation model to predict body temperature, blood pressure, and blood flow rate. Our model consists of a “physical model” that simulates the phenomena of heat and blood transfer based on the physical properties of the human body, and an “autonomic regulation model” that simulates the phenomena in which the physical properties of the human body are regulated by the activity of the autonomic nervous system. The physical model consists of a “thermal network (TNW) model” that simulates heat flow and body temperature, and a “cardiovascular (CV) model” that simulates blood flow and blood pressure. In this study, we focused only on developing the physical model. In addition, based on experimental data from a recent study, parameter settings were derived to simulate an average Japanese male in their 20s. By applying the derived parameter settings to the physical model developed in this study, we confirmed that the calculated body temperature, blood flow rate, and blood pressure under a thermally neutral and supine condition reproduced the experimental values well.

**Supplementary Information:**

The online version contains supplementary material available at 10.1007/s00484-025-03072-6.

## Introduction

Inappropriate thermal environments can cause health problems. In hot environments, not only does the problem of hyperthermia arise, but problems of a decrease in orthostatic tolerance (Rowell 1986) and the occurrence of heat syncope (Ministry of the Environment of Japan 2022) also arise due to decreases in blood pressure (BP) and blood flow rate (BFR) in the brain. The decreases in BP and BFR in the brain are caused by vasodilation and increased BFR in the skin and extremities for thermoregulation. In cold environments, not only does the problem of hypothermia arise, but the problem of an increase in BP also arises due to vasoconstriction and reduced BFR in the skin and extremities for thermoregulation. The increase in BP can lead to the development of cerebrovascular disease in patients with hypertension (Umemura et al. 2019). In addition, rapid fluctuations in BP due to sudden changes in ambient temperature can cause cerebral stroke and heart attack (Tochihara 2012a, b). Thus, changes in body temperature (BT), BP, and BFR can directly trigger thermal health problems.

BP and BFR are also important for correctly assessing the risks of hyperthermia and hypothermia. It is well known that BFR plays an important role in maintaining the core temperature of the body; however, it is not solely related to BT regulation. BFR can change due to postural changes, for example, from a seated to a standing position, because they induce changes in BP distribution in the body (Rowell 1986). It can also change due to exercise, which requires an increase in oxygen supply to various parts of the body (Rowell 1986). Therefore, BFR is not necessarily controlled in the same manner under the same BT conditions, and the effects of posture and exercise on BP and BFR compete with BFR regulation to maintain BT. Consequently, the actual BFR is realized by compromising these factors.

Thus, BT, BP, and BFR are influenced not only by the thermal environment but also by individual behavior. Moreover, the physical constitution and predisposition of individuals are also important influencing factors. The risk of thermal health problems varies depending on the combination of these influencing factors. Human-body simulations are useful for assessing the risks brought about by various and complicated combinations of these factors.

Various human-body simulation models have been developed (Katic et al. 2016). The Stolwijk model (Stolwijk 1971) is the most representative model that considers the shape of the human body and can predict temperature distribution throughout the body. Autonomic thermoregulatory functions of the human body, such as vasomotion, sweating, and shivering heat production, have also been modeled. In recent years, advanced models, such as those proposed by Tanabe (Tanabe et al. 2001, 2006; Kobayashi et al. 2013; Takahashi et al. 2021), Huizenga and Zhang (Huizenga et al. 2001; Zhang et al. 2001), Fiala et al. 1999, 2001), have become well known. These models are called thermophysiological models, and are often used to evaluate human thermal stress in indoor and outdoor environments. However, these models primarily focus on predicting the BT, and their validity for predicting the BFR has not been sufficiently tested. This is because there was a paucity of available measurement data for the BFR. In addition, these models do not have the ability to predict BP.

Simulation models for BP and BFR have been developed to understand the physical phenomena in the cardiovascular system and support the diagnosis of cardiovascular diseases, such as arterial and aortic stenosis. The earliest studies were by Noordergraaf et al. (1963) and Westerhof et al. (1969). They proposed an analog computer model in which the systemic arterial tree was replaced with an electrical circuit. With reference to their model, an analytical lumped-parameter model (Avolio 1980) and one-dimensional (1-D) numerical fluid dynamics models (Schaarf and Abbrecht 1972; Stergiopulos et al. 1992; Olufsen et al. 2000) of the arterial tree were developed. Formaggia et al. (2006) and Mynard and Nithiarasu (2008) coupled a 1-D numerical fluid dynamics model of the arterial tree with a lumped-parameter model of the heart. Liang et al. (2009) completed the entire model of the closed-loop structure of the cardiovascular system. They coupled a 1-D numerical fluid dynamics model of the arterial tree with a lumped-parameter model of other elements, such as the peripheral circulations, venous system, and cardiopulmonary system. Thus, simulation technology for the cardiovascular system has already been developed outside thermophysiological modeling.

A few previous studies combined the aforementioned thermophysiological and cardiovascular models. Ghaddar and her colleagues (Salloum et al. 2007; Karaki et al. 2013; Rida et al. 2014) generated a model that combined the Stolwijk and Avolio models. Coccarelli et al. (2016, 2018) developed a model that combined the Fiala model and Mynard and Nithiarasu model. Liu et al. (2023) created a model that combined the Stolwijk model with a simple lumped-parameter cardiovascular model. However, the validity of their BFR predictions remains uncertain because their BFR predictions are essentially based on existing thermophysiological models. In addition, coupled cardiovascular models do not consider the venous system, or the cardiovascular model is overly simplified, making it impossible to make valid BP predictions for various conditions with different postures and thermal environments. Therefore, we aimed to develop a more realistic human-body simulation model that simultaneously predicts BT, BP, and BFR by coupling thermophysiological and cardiovascular models.

In human-body simulations, it is reasonable to treat the heat and blood transfer phenomena based on the physical properties of the human body separately from the phenomena in which these physical properties are regulated by the activities of the autonomic nervous system. This is a common concept in conventional thermophysiological models, which consist of a controlled system and a controlling system (Stolwijk 1971), or a passive system and an active system (Fiala et al. 1999, 2001). This study focused on developing a model that simulates the transfer of heat and blood based on the physical properties of the human body. The other model, which simulates phenomena in which the physical properties of the human body are regulated by the activity of the autonomic nervous system, will be addressed in the next paper. In addition, standard parameter settings were derived in this study. Although the ultimate goal of our present and future studies is to provide fine-tuned risk assessments according to differences in personal characteristics, it must be impossible to prepare tailor-made personalized parameter settings for every individual. Therefore, we aimed to prepare standard model parameter settings for personalizing later according to age, pre-existing diseases, etc. We chose an average Japanese male in their 20s as the standard person. This is because detailed experimental data on BFRs for this type of person were recently obtained (Goto et al. 2024) and could be used as reference data for deriving the parameter settings.

## Structure and mathematical modeling of human simulation

### Overall structure of the human simulation model

**Fig. 1:**
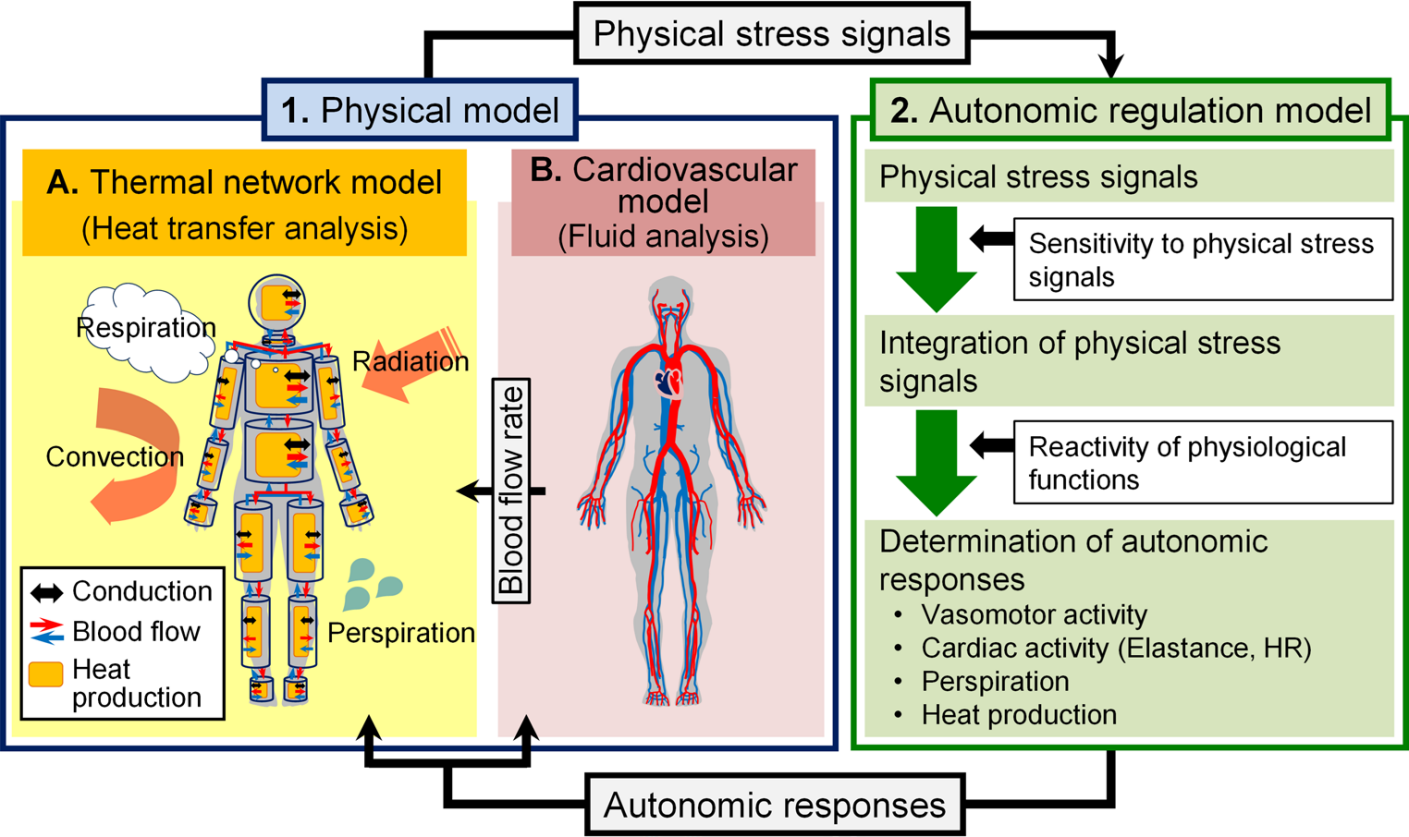
Overall structure of the human simulation model

The overall structure of the proposed model is illustrated in Fig. 1. This model consists of a “physical model” that simulates the phenomena of heat and blood transfer based on the physical properties of the human body (i.e., including the phenomena of temperature and blood pressure propagation) and an “autonomic regulation model” that simulates the phenomena in which the physical properties of the human body are regulated by autonomic nervous system activity. The physical model consists of a “thermal network (TNW) model” that simulates heat flow and body temperature, and a “cardiovascular (CV) model” that simulates BFR and BP. The BFR calculated in the CV model was used as the BFR in the TNW model. This study only dealt with the physical model.

### Thermal network (TNW) model

**Fig. 2:**
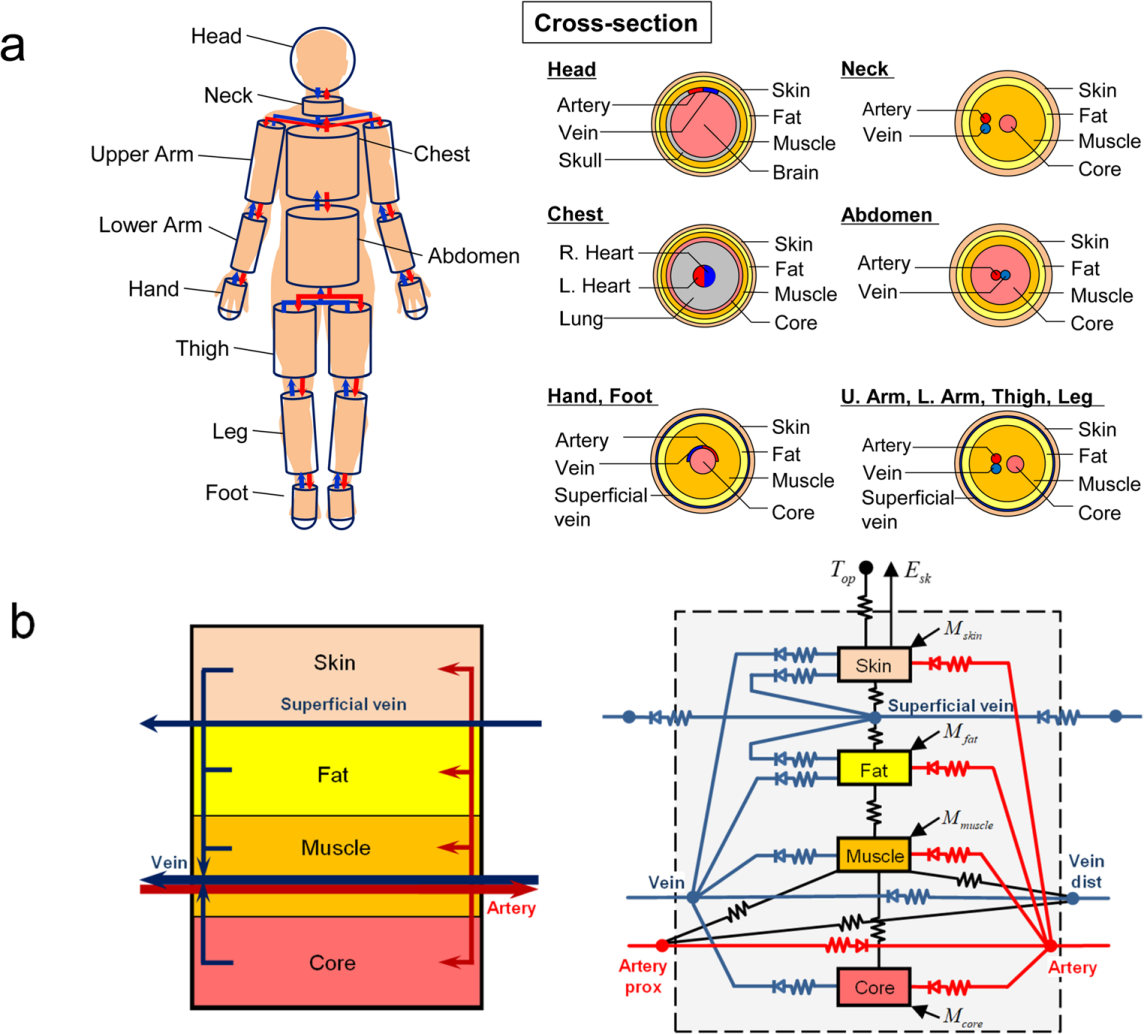
Schematic diagram of the thermal network model **a**: segmentation and decomposition of the body; **b**: configuration of the thermal network within an example segment (leg)

The TNW model was developed based on the Stolwijk model (1971). In this model, the human body is divided into 16 segments, each of which is subdivided into four compartments: core, muscle, fat, and skin (Fig. 2a). However, the head is divided into the brain, skull, muscle, fat, and skin, and the chest is divided into the left heart, right heart, lung, core, muscle, fat, and skin. Arteries and veins are located in all segments, and superficial veins are located in the limb segments. In this study, each of the abovementioned compartments and blood vessels is referred to as a node.

Each compartment transfers heat to and from the adjacent compartments in the same segment by conduction (Fig. 2b). Blood flows into each compartment from the artery of the same segment, and blood flows into the vein of the same segment from the compartment. For the limb compartments, part of the blood from the compartments flows into the superficial vein. The heat balance equation for compartment *I* is expressed as

1 $$\begin{array}{c}C_{I}\frac{dT_{I}}{dt}=M_{I}+\sum_{J}\left\{\left({TC}_{I,J}+\rho c Q_{I,J}\right)\left(T_{J}-T_{I}\right)\right\}\\-C_{res}-E_{res}-E_{sk,I}+q_{I,ar}+q_{I,ve}\end{array}$$

where *Q*_*I*,*J*_ is the BFR from node *J* to node *I*, and If *Q*_*I*,*J*_ is negative, it is set to zero. Sensible heat loss *C*_*res*_ and latent heat loss *E*_*res*_ due to respiration are included in the equation only when node *I* is the lung, and latent heat loss from the skin *E*_*sk*,*I*_ is included in the equation only when node *I* is the skin. If node *J* is an artery or vein and is adjacent to compartment *I*, the thermal conductance *TC*_*I*,*J*_ between *I* and *J* is set to zero, and *q*_*I*,*ar*_ and *q*_*I*,*ve*_, described below, are included in the equation.

The Stolwijk model ignores heat exchange through blood vessels between the heart and body segments, whereas the model in this study considers heat exchange through blood vessels. The artery node of each segment receives blood from the artery node of the neighboring body segment on the proximal side. However, the artery nodes of the abdomen, neck, and upper arm segments receive blood from the left heart compartment of the chest segment. The vein and superficial vein nodes of each segment receive blood not only from the compartments of the individual segments but also from the vein and superficial vein nodes of the neighboring body segments on the distal side. In addition, blood from the vein and superficial vein nodes of the thigh segments flows into the vein node of the abdomen, and blood from the vein nodes of the neck, abdomen, upper arm segments, and superficial vein nodes of the upper arm segments flows into the right heart compartment of the chest segment. Blood flows from the right heart to the left heart through the lung compartment.

Except for the head, hands, and feet segments, heat exchange in the artery and vein with adjacent compartments is considered as piston flow, and heat gains from adjacent compartment *I* in the artery and vein are calculated using the following equations:

2 $$q_{ar,I}=\rho c Q_{ar}\left\{1-\mathrm{exp}\left(-\frac{{TC}_{ar,I}}{\rho c Q_{ar}}\right)\right\}\left(T_{I}-T_{ar\_prox}\right)=-q_{I,ar}$$

3 $$q_{ve,I}=\rho c Q_{ve}\left\{1-\mathrm{exp}\left(-\frac{{TC}_{ve,I}}{\rho c Q_{ve}}\right)\right\}\left(T_{I}-T_{ve\_dist}\right)=-q_{I,ve}$$

where *Q*_*ar*_ and *Q*_*ve*_ are the BFRs in the artery and vein, respectively. *TC*_*ar*,*I*_ and *TC*_*ve*,*I*_ are the composites of the convective heat transfer coefficient in the vessel and thermal conductance between the vessel and adjacent compartment.

Counterflow heat exchange between the artery and vein was also considered. Based on the same theory as for the heat exchangers in HVAC systems, this heat transfer can be calculated using the following equation:

4 $$\begin{array}{c}q_{ar,ve}=\rho c Q_{ve}\frac{1-\mathrm{exp}\left\{-\frac{{TC}_{ar,ve}}{\rho c Q_{ve}}\left(1-\frac{Q_{ve}}{Q_{ar}}\right)\right\}}{1-\frac{Q_{ve}}{Q_{ar}}\mathrm{exp}\left\{-\frac{{TC}_{ar,ve}}{\rho c Q_{ve}}\left(1-\frac{Q_{ve}}{Q_{ar}}\right)\right\}}\\\times\left(T_{ve\_dist}-T_{ar\_prox}\right)=-q_{ve,ar}\end{array}$$

where *TC*_*ar*,*ve*_ is the composite of the convective heat transfer coefficients in the artery and vein as well as the thermal conductance of the tissue between the artery and vein.

If we neglect the time-derivative term and the interaction of heat transfer in Eqs. (2)–(4), the heat balance of the artery and vein can be expressed as follows:

5 $$\rho c Q_{ar}\left(T_{ar\_prox}-T_{ar}\right)+q_{ar,adj}+q_{ar,ve}=0$$

6 $$\begin{array}{c}\rho cQ_{ve}\left(T_{ve\_dist}-T_{ve}\right)+\sum_J\left\{\rho cQ_{ve,J}\left(T_J-T_{ve}\right)\right\}\\\:+q_{ve,adj}+q_{ve,ar}=0\end{array}$$

where *q*_*ar*,*adj*_ and *q*_*ve*,*adj*_ represent the heat gains from the adjacent compartments of the artery and vein, respectively.

The artery and vein nodes of the head, hands, feet, and all superficial vein nodes were assumed to be perfect mixing blood pools. The heat balance equation for these vessels is the same as that in Eq. (1). The temperatures of the individual body nodes can be calculated by discretizing Eq. (1) with respect to time, and solving Eqs. (1), (5), and (6) simultaneously.

### Cardiovascular (CV) model

Our CV model was developed based on the model proposed by Liang et al. (2009). This model consists of a one-dimensional model (1-D model) that reproduces the major arteries and a lumped-parameter model (0-D model) that reproduces the peripheral circulation, vena cava, heart, and pulmonary circulation.

**Fig. 3:**
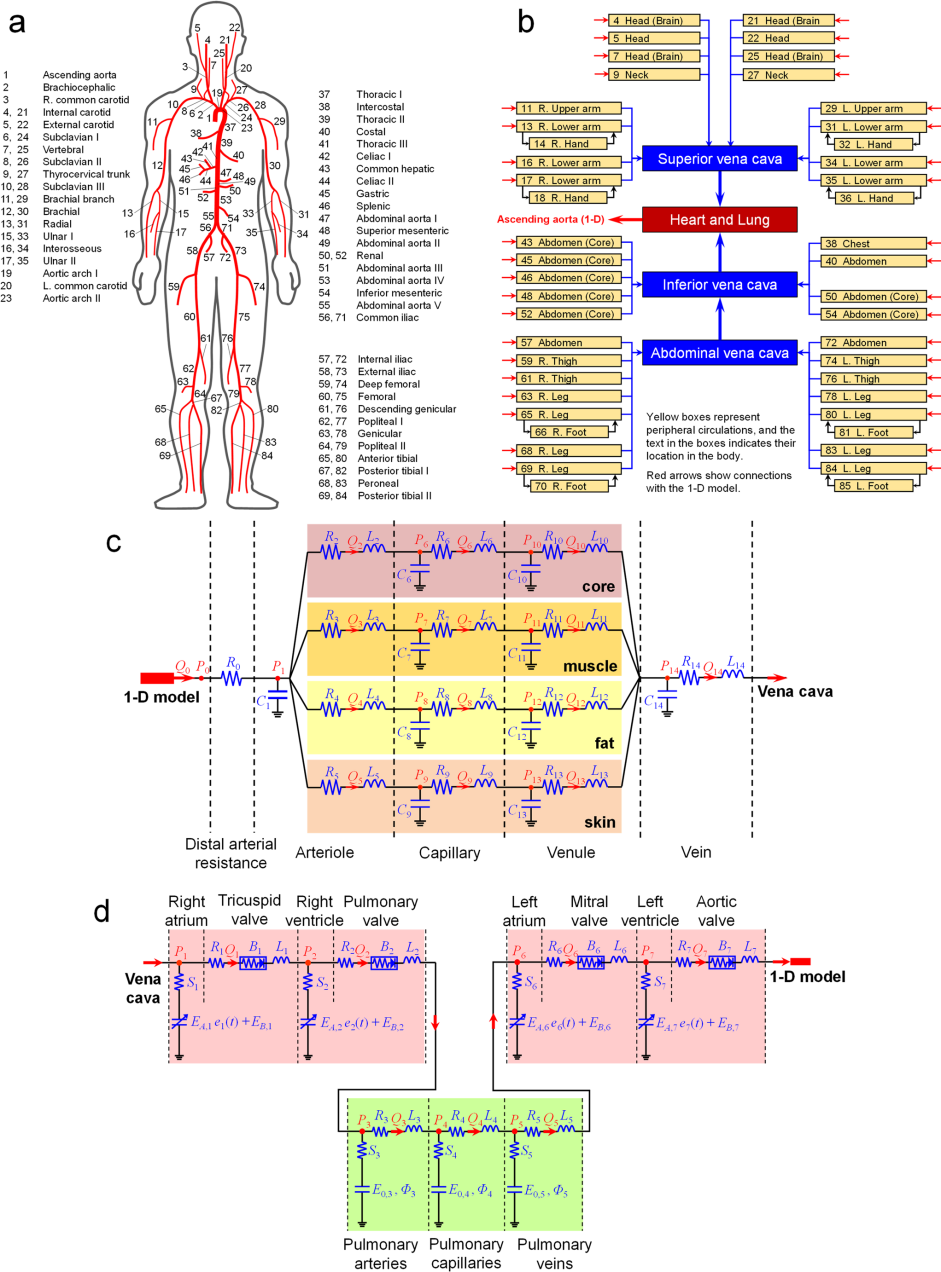
Schematic diagram of the cardiovascular model **a**: overview of the 1-D model; **b**: overview of the 0-D model, **c**: configuration of a typical peripheral circulation; **d**: configuration of the heart and pulmonary circulation

The 1-D model consists of 77 major arteries throughout the body. The ID numbers and names of the arteries comprising the 1-D model are shown in Fig. 3a. The brachial branch (No.11, 29), intercostal (No.38), costal (No.40), and genicular (No.63, 78) arteries were simplified from multiple arteries that run in a complex manner and replaced by a single artery. The governing equations for the 1-D model are as follows:

7 $$\frac{\partial A}{\partial t}+\frac{\partial Q}{\partial x}=0$$

8 $$\frac{\partial Q}{\partial t}+\frac{\partial}{\partial x}\left(\frac{Q^2}{A}\right)+\frac{A}{\rho}\frac{\partial}{\partial x}\left(P+\rho g H\right)+\frac{2\pi r\nu}{\delta}\frac{Q}{A}=0$$

9 $$P=\frac{Eh}{r_{0}\left(1-\sigma^{2}\right)}\left(\sqrt{\frac{A}{A_{0}}}-1\right)$$

Eqs. (7) and (8) represent the mass and momentum conservation equations, respectively. Eq. (9) is a state equation that expresses the relationship between BP and the cross-sectional area of arteries. In Eq. (8), the momentum equation of the Liang model is modified so that the effect of gravity can be taken into account.

To calculate the BFR and BP for each artery, Eqs. (7) and (8) are solved using the finite difference method after substituting Eq. (9) into Eq. (8). In addition, the conservation of BFR and continuity of BP are satisfied at each connection of the arteries, that is, at each bifurcation in the arterial tree. The boundary conditions of the 1-D model, that is, the BFR at the root of the arterial tree and the BPs at the terminals of the arterial tree, can be provided by the calculation results of the 0-D model.

The 0-D model expresses the entire cardiovascular system, other than the arteries in the 1-D model, as electrical circuits. As shown in Fig. 3b, the terminal arteries of the 1-D model were connected to the peripheral circulations of the 0-D model. These peripheral circulations connect to the superior, inferior, or abdominal vena cavae and then to the cardiopulmonary system. Figure 3c shows the configuration of the typical peripheral circulation in our 0-D model. In the Liang model, there is only one route in the peripheral circulation. However, in our model, the peripheral circulation is divided into four routes (core, muscle, fat, and skin) for consistency with the TNW model. Each capillary and venule of each route and the vein into which the route rejoins are represented by a basic unit consisting of three elements (compliance *C*, viscosity resistance *R*, and inertial resistance *L*) that represent the vascular characteristics. However, for the arterioles, the four bifurcated units share a single compliance. The three venae cavae connecting downstream of the veins are represented by the same basic units as those of the veins. The BFR and BP at each site in the peripheral circulation and vena cava are calculated using the following equations:

10 $$\frac{dV_{i}}{dt}=Q_{up}-Q_{i}$$

11 $$P_{i}-P_{dw}=Q_{i}R_{i}+L_{i}\frac{dQ_{i}}{dt}+\rho g\left(H_{dw}-H_{i}\right)$$

12 $$P_{i}=\frac{V_{i}}{C_{i}}$$

Eqs. (10) and (11) represent the mass and momentum conservation equations, respectively. Eq. (12) is the state equation for the relationship between the BP and vascular volume.

The heart and pulmonary circulation of the 0-D model are illustrated in Fig. 3d. Here, elastance *E* is used instead of compliance *C*, and the viscoelastic resistance *S* and pressure-loss coefficient *B* of the heart valve are given as parameters. Elastance *E* refers to the ratio of the pressure change to the volume change (*dP*/*dV*), and elastance *E* and compliance *C* are the inverse of each other. The mass conservation equation in the heart is the same as that in peripheral circulation, Eq. (10), but the momentum and state equations are different as follows:

13 $$P_{i}-P_{dw}=Q_{i}R_{i}+{Q_{i}}^2 B_{i}+L_{i}\frac{dQ_{i}}{dt}$$

14 $$P_{i}=\left(E_{A,i}e_{i}\left(t\right)+E_{B,i}\right)V_{i}+S_{i}\frac{dV_{i}}{dt}$$

where *e*_*i*_(*t*) is a function of time representing the contraction and relaxation of the heart. In the case of the atria:

15 $$e_{i}\left(t\right)=\left\{\begin{array}{l}0.5\left\{1+\mathrm{cos}\left(\pi \frac{T+1-\left(T_{cs,i}+T_{cp,i}\right)}{T_{rp,i}}\right)\right\}\quad\left(0<T\le T_{cs,i}+T_{cp,i}+T_{rp,i}-1\right)\\0\quad\left(T_{cs,i}+T_{cp,i}+T_{rp,i}-1<T\le T_{cs,i}\right)\\0.5\left\{1-\mathrm{cos}\left(\pi \frac{T-T_{cs,i}}{T_{cp,i}}\right)\right\}\quad\left(T_{cs,i}<T\le T_{cs,i}+T_{cp,i}\right)\\0.5\left\{1+\mathrm{cos}\left(\pi \frac{T-\left(T_{cs,i}+T_{cp,i}\right)}{T_{rp,i}}\right)\right\}\quad\left(T_{cs,i}+T_{cp,i}<T\le 1\right)\end{array}\right.$$

In the case of the ventricles:

16 $$e_{i}\left(t\right)=\left\{\begin{array}{l}0\quad\left(0<T\le T_{cs,i}\right)\\0.5\left\{1-\mathrm{cos}\left(\pi \frac{T-T_{cs,i}}{T_{cp,i}}\right)\right\}\quad\left(T_{cs,i}<T\le T_{cs,i}+T_{cp,i}\right)\\0.5\left\{1+\mathrm{cos}\left(\pi \frac{T-\left(T_{cs,i}+T_{cp,i}\right)}{T_{rp,i}}\right)\right\}\quad\left(T_{cs,i}+T_{cp,i}<T\le T_{cs,i}+T_{cp,i}+T_{rp,i}\right)\\0\quad\left(T_{cs,i}+T_{cp,i}+T_{rp,i}<T\le 1\right)\end{array}\right.$$

For the pulmonary circulation, Eqs. (10) and (11) are used as the mass and momentum equations, respectively, and the following equation is used as the state equation:

17 $$P_{i}=E_{0,i}\varPhi_{i}\mathrm{exp}\left(\frac{V_{i}}{\varPhi_{i}}\right)+S_{i}\frac{dV_{i}}{dt}$$

The BFRs and BPs in the 0-D model can be calculated by substituting the state equation into the momentum conservation equation, discretizing the mass and momentum conservation equations with respect to time, and numerically solving them. The boundary conditions of the 0-D model, that is, the BFRs at the inlets of the peripheral circulations and BP at the outlet of the aortic valve, can be obtained from the calculation results of the 1-D model.

## Derivation of standard parameter settings for the physical model

### Parameter settings of TNW model

The parameters to be provided in the TNW model are the heat capacity and metabolic rate of each node and the thermal conductance between nodes. To reproduce the average Japanese male in their 20s according to the best available information, these values were derived based on the voxel model of Nagaoka et al. (2004). Nagaoka et al. provided voxel model data with 2 mm spatial resolution and 55 types of tissues and organs based on MRI data of volunteers, matching the average height and weight of adult men and women. In this study, a male model (TARO) was used to derive information on body configuration. The height and weight of the male model (TARO) are 172 cm and 65 kg, respectively. The surface area and volume of each segment and the volume of each compartment obtained using this voxel model are listed in Table S1 in the Supplementary Material.

The heat capacity and metabolic rate were calculated based on the volume of each component, as well as the volumetric specific heat, density, and basal metabolic rate per unit mass of each tissue or organ (Table S2, Supplementary Material). The calculated heat capacity and metabolic rate of each node are listed in Table 1. For the arteries and veins, the heat capacity was not set because the heat balance was calculated using Eqs. (5) and (6), neglecting the time-derivative term. The heat capacity of superficial veins was also ignored in this study.

**Table 1:** Heat capacity, basal metabolic rate, and simplified geometry of each node in the TNW model

Segment	Node	Heat capacity [J/K]	Basal metabolic rate [W]	Segment length [m]	Compartment or vessel radius [m]	Segmental sum of vessel surface area [m^2^]
Head	Brain	5523.3	17.470	–	0.070	
	Skull	4208.6	1.109		0.089	
	Muscle	2627.6	0.486		0.097	
	Fat	1342.3	0.127		0.103	
	Skin	958.9	0.148		0.105	
	Artery^*1^	–	–		–	–
	Vein^*1^	–	–		–	–
Neck	Core	406.4	0.095	0.065	0.025	
	Muscle	1200.0	0.222		0.047	
	Fat	282.3	0.027		0.054	
	Skin	173.4	0.027		0.056	
	Artery^*2^	–	–		0.004	0.016
	Vein^*3^	–	–		0.004	0.019
Chest	Right heart	801.4	4.596	0.354	–^*4^	
	Left heart	801.4	4.596		–^*4^	
	Lung	7383.1	1.156		0.058	
	Core	8924.9	2.040		0.079	
	Muscle	14050.1	2.601		0.099	
	Fat	3266.9	0.309		0.106	
	Skin	1815.2	0.280		0.108	
Abdomen	Core	26520.1	28.286	0.530	0.075	
	Muscle	27446.6	5.082		0.101	
	Fat	7009.4	0.663		0.111	
	Skin	2829.2	0.436		0.113	
	Artery^*2^	–	–		0.007	0.046
	Vein^*3^	–	–		0.008	0.055
Upper arm (right or left)	Core	587.0	0.214	0.288	0.016	
	Muscle	3892.2	0.721		0.038	
	Fat	1006.3	0.095		0.045	
	Skin	640.8	0.099		0.047	
	Artery^*2^	–	–		0.003	0.010
	Vein^*3^	–	–		0.004	0.024
Lower arm (right or left)	Core	305.0	0.111	0.176	0.015	
	Muscle	1929.3	0.357		0.034	
	Fat	371.0	0.035		0.039	
	Skin	343.7	0.053		0.041	
	Artery^*2^	–	–		0.002	0.006
	Vein^*3^	–	–		0.002	0.014
Hand (right or left)	Core	193.2	0.070	0.257	0.010	
	Muscle	449.1	0.083		0.016	
	Fat	270.4	0.026		0.020	
	Skin	293.4	0.045		0.022	
	Artery^*2^	–	–		4.5 × 10^− 4^	0.003
	Vein^*3^	–	–		5.4 × 10^− 4^	0.004
Thigh (right or left)	Core	1000.4	0.365	0.254	0.023	
	Muscle	11748.2	2.175		0.068	
	Fat	2879.3	0.272		0.080	
	Skin	987.1	0.152		0.082	
	Artery^*2^	–	–		0.003	0.011
	Vein^*3^	–	–		0.003	0.013
Leg (right or left)	Core	1035.6	0.378	0.286	0.022	
	Muscle	5176.2	0.958		0.045	
	Fat	1244.4	0.118		0.053	
	Skin	740.7	0.114		0.055	
	Artery^*2^	–	–		0.002	0.012
	Vein^*3^	–	–		0.002	0.028
Foot (right or left)	Core	703.3	0.257	0.288	0.018	
	Muscle	843.4	0.156		0.023	
	Fat	660.1	0.062		0.030	
	Skin	484.4	0.075		0.032	
	Artery^*2^	–	–		4.4 × 10^− 4^	0.007
	Vein^*3^	–	–		5.3 × 10^− 4^	0.008

*1 The actual arteries and veins of the head run complicatedly inside and outside the skull and are difficult to simplify. Therefore, the radius and surface area were not set, and the thermal conductance between the artery/vein and other nodes was neglected.

*2 The radius and surface area of the artery in each segment were given as the average radius and sum of the surface areas of the arteries of the CV model considered to be in that segment. For the hands and feet, the radius and surface area of the arteries were provisionally set to 1/4 of the adjacent segment (lower arms and legs) and 20% of the surface area of the core (bone), respectively, because no quantitative reference information could be found.

*3 The radius and surface area of the vein were set to 1.2 times those of the artery, referring to Levick (2010). However, the surface area in the upper arms, lower arms, and legs was assumed to be 2.4 times that of the artery because two veins run parallel to one artery in these body regions.

*4 The radius of the heart was not set because only the heat transfer between the heart and lung by blood flow was considered, and the heat transfer between the heart and lung by conduction was neglected.

To determine the thermal conductance between the nodes, we simplified the geometries of the segments, such as the head to a sphere, the hand and foot to a combined cylinder-hemisphere, and the other parts to cylinders. Table 1 presents the simplified geometries (length and radius of each segment and node) obtained so that the surface area and volume of each segment and the volume of each compartment were consistent with those in Table S1 in the Supplementary Material. Tables S3 and S4 in the Supplementary Material provide the specific calculation methods. The calculated thermal conductance values between the nodes are listed in Table S5 in the Supplementary Material.

### Parameter settings of CV model

The parameters to be provided in the 1-D model are the length, radius at the reference state of each artery, and thickness and Young’s modulus of the wall of each artery. These parameters were set based on previous studies (Table 2). The length of the arteries was given with reference to Westerhof et al. (1969), which is the basis for many cardiovascular models, and similar values have been used in several recent studies (Olufsen et al. 2000; Wang and Parker 2004; Liang et al. 2009). In Westerhof, a human with a height of 175 cm and weight of 75 kg was assumed, which is almost the same as the voxel model of Nagaoka et al. (2004) with respect to height, but slightly heavier with respect to weight. For the radii of arteries, we referred to a recent study by Liang et al. (2009). Different researchers have provided different values for vascular wall thickness *h* and Young’s modulus *E*. These values are often adjusted by examining the calculated BP waveforms, and there is no clear rationale for setting these values. Therefore, the relative thickness *h*/*r*_0_ and Young’s modulus *E* of the vessel wall were given with reference to Westerhof et al. (1969).

**Table 2:** Characteristics of each artery in the CV model

ID No.	Name of artery	Length *l* [m]^*1^	Proximal radius *r*_0,prox_ [m]^*2^	Distal radius *r*_0,dist_ [m] ^*2^	Relative wall thickness *h*/*r*_0_^*3^	Young’s modulus *E* [Pa] ^*3^
1	Ascending aorta	0.040	0.01525	0.01405	0.112	400,000
2	Brachiocephalic	0.034	0.00650	0.00620	0.137	400,000
3	R. common carotid	0.177	0.00400	0.00370	0.167	400,000
4, 21	Internal carotid	0.177	0.00300	0.00275	0.192	800,000
5, 22	External carotid	0.177	0.00200	0.00200	0.232	800,000
6, 24	Subclavian I	0.034	0.00425	0.00407	0.161	400,000
7, 25	Vertebral	0.148	0.00200	0.00200	0.232	800,000
8, 26	Subclavian II	0.015	0.00407	0.00399	0.163	400,000
9, 27	Thyrocervical trunk	0.100	0.00100	0.00100	0.342	800,000
10, 28	Subclavian III	0.170	0.00399	0.00317	0.173	400,000
11, 29	Brachial branch	0.150	0.00120	0.00120	0.308	800,000
12, 30	Brachial	0.235	0.00317	0.00230	0.198	400,000
13, 31	Radial	0.235	0.00175	0.00140	0.266	800,000
15, 33	Ulnar I	0.067	0.00215	0.00215	0.223	800,000
16, 34	Interosseous	0.079	0.00100	0.00100	0.342	1,600,000
17, 35	Ulnar II	0.171	0.00203	0.00180	0.238	800,000
19	Aortic arch I	0.020	0.01405	0.01349	0.113	400,000
20	L. common carotid	0.208	0.00400	0.00370	0.167	400,000
23	Aortic arch II	0.039	0.01349	0.01246	0.114	400,000
37	Thoracic I	0.080	0.01246	0.01058	0.117	400,000
38	Intercostal	0.073	0.00300	0.00300	0.187	400,000
39	Thoracic II	0.060	0.01058	0.00936	0.120	400,000
40	Costal	0.073	0.00233	0.00233	0.214	400,000
41	Thoracic III	0.060	0.00936	0.00828	0.124	400,000
42	Celiac I	0.020	0.00350	0.00300	0.181	400,000
43	Common hepatic	0.066	0.00275	0.00250	0.201	400,000
44	Celiac II	0.020	0.00300	0.00250	0.196	400,000
45	Gastric	0.071	0.00175	0.00150	0.261	400,000
46	Splenic	0.063	0.00200	0.00200	0.232	400,000
47	Abdominal aorta I	0.045	0.00828	0.00755	0.128	400,000
48	Superior mesenteric	0.059	0.00400	0.00350	0.169	400,000
49	Abdominal aorta II	0.015	0.00755	0.00732	0.130	400,000
50, 52	Renal	0.032	0.00275	0.00275	0.196	400,000
51	Abdominal aorta III	0.015	0.00732	0.00710	0.131	400,000
53	Abdominal aorta IV	0.065	0.00710	0.00622	0.135	400,000
54	Inferior mesenteric	0.050	0.00200	0.00175	0.241	400,000
55	Abdominal aorta V	0.060	0.00622	0.00550	0.141	400,000
56, 71	Common iliac	0.058	0.00400	0.00370	0.167	800,000
57, 72	Internal iliac	0.045	0.00200	0.00200	0.232	800,000
58, 73	External iliac	0.144	0.00370	0.00314	0.176	800,000
59, 74	Deep femoral	0.126	0.00255	0.00186	0.221	1,600,000
60, 75	Femoral	0.254	0.00314	0.00275	0.189	800,000
61, 76	Descending genicular	0.135	0.00197	0.00144	0.255	1,600,000
62, 77	Popliteal I	0.137	0.00275	0.00275	0.196	1,600,000
63, 78	Genicular	0.050	0.00119	0.00119	0.311	1,600,000
64, 79	Popliteal II	0.052	0.00275	0.00275	0.196	1,600,000
65, 80	Anterior tibial	0.343	0.00175	0.00175	0.250	1,600,000
67, 82	Posterior tibial I	0.025	0.00250	0.00250	0.206	1,600,000
68, 83	Peroneal	0.318	0.00130	0.00130	0.295	1,600,000
69, 84	Posterior tibial II	0.322	0.00175	0.00175	0.250	1,600,000

*1 The length of the arteries was given with reference to Westerhof et al. (1969). For arteries not addressed by Westerhof, the length was estimated by referring to other studies (Avolio 1980; Liang et al. 2009) and human anatomy charts (Otani and Kawahara 2012; Tortora and Nielsen 2009). For the brachial branch (No.11, 29), intercostal (No.38), costal (No.40), and genicular (No.63, 78) arteries, the length was given expediently, because these were modeled as substitutes for multiple arteries running in a complex manner and could not be given accurately.

*2 The radii of the arteries were given with reference to Liang et al. (2009). For arteries not addressed by Liang et al., the radii were estimated by referring to Avolio (1980) or by actual measurements (Lorbeer et al. 2018; Szpinda 2005). Expedient values were set for the radii of the brachial branch, costal, and genicular arteries for the same reason as the length.

*3 The relative thickness and Young’s modulus of the vessel wall were given with reference to Westerhof et al. (1969). For arteries not addressed by Westerhof, the values were set by referring to similar arteries addressed by Westerhof.

The 0-D model requires parameters for the peripheral circulations, venae cavae, heart, and pulmonary circulation. These parameter values, particularly for the peripheral circulations and heart, should vary owing to autonomic regulation in response to thermal and postural conditions. This study considered only the state of the cardiovascular system under the condition of minimal autonomic regulation because autonomic regulation was beyond the scope of this study. We attempted to derive the parameter settings to reproduce the BFR and BP measured under the thermally neutral and supine condition (baseline condition) (Goto et al. 2024) by modifying the parameter values of Liang’s model. In addition, we primarily modified the parameter values for the peripheral circulations, because the BFR distribution in the body is mainly determined by those. The parameter values for the venae cavae, heart, and pulmonary circulation were not modified (Tables S6 and S7, Supplementary Material), but only the heart rate was slightly modified (from 60 to 62 beats/min).

Regarding the parameters of peripheral circulations, Liang et al. (2009) set the magnitude of viscosity resistance *R* of the distal arterial end, arteriole, capillary, venule, and vein connected to each artery at ratios of 17%, 54%, 21%, 6.5%, and 1.5% to the sum of those viscosity resistances (total viscosity resistance *R*_*T*_), respectively. In addition, they set the magnitude of the inertial resistance *L* of the arteriole, capillary, venule, and vein to be proportional to the square root of the total viscosity resistance *R*_*T*_, and the magnitude of the compliance *C* of the arteriole, capillary, venule, and vein to be inversely proportional to the total viscosity resistance *R*_*T*_. Therefore, we adjusted the *RLC* values while maintaining these relationships and reproducing the arterial BFRs measured under the baseline condition (Goto et al. 2024). However, we changed the ratio of *R* such that the arteriole had 90% of the *R*_*T*_, and the remaining 10% was allocated to the distal arterial end, capillary, venule, and vein. This is because the ratio of the viscosity resistance of the arteriole to the *R*_*T*_ was estimated to be much larger, considering that the BFR is mainly controlled by the vasomotor activity of the arteriole, that skin BFR in hot environments can be ten times greater than that in a neutral environment, and that muscle BFR during exercise can be ten times greater than that at rest (Rowell 1986).

Furthermore, the peripheral circulation in our model differed from that in the Liang model – our model has four routes, whereas Liang’s model has only a single route, as described in the "Cardiovascular (CV) model" section. Therefore, we decomposed the single-route *RLC* circuit into a four-route *RLC* circuit. The decomposed *RLC* elements of the peripheral circulation were adjusted to reproduce a realistic BFR distribution, which was determined based on the measurements (Table S8, Supplementary Material), while maintaining the BPs of the capillary and venule before and after the decomposition. Consequently, the peripheral circulation parameter values were determined (Table S9, Supplementary Material).

### Simulation results by the developed physical model

Using the model parameter settings derived as described above, we performed a simulation to reproduce the experiment by Goto et al. (2024) under the baseline condition. The TNW model was solved using an implicit method with a computational time interval of 10 s. The CV model was solved with a time interval of 0.0001 s, the 1-D model was solved using the two-step Lax–Wendroff method, and the 0-D model was solved using the Runge–Kutta method with a fourth-order accuracy. The average BFR per heartbeat, calculated by the CV model, was passed to the TNW model every 10 s and used as the BFR in the TNW model.

The ambient temperature and relative humidity were set at 28 °C and 40%, respectively. Other boundary conditions, such as heat transfer coefficients, were given based on our measurements using a thermal manikin (Table S10, Supplementary Material).

**Fig. 4:**
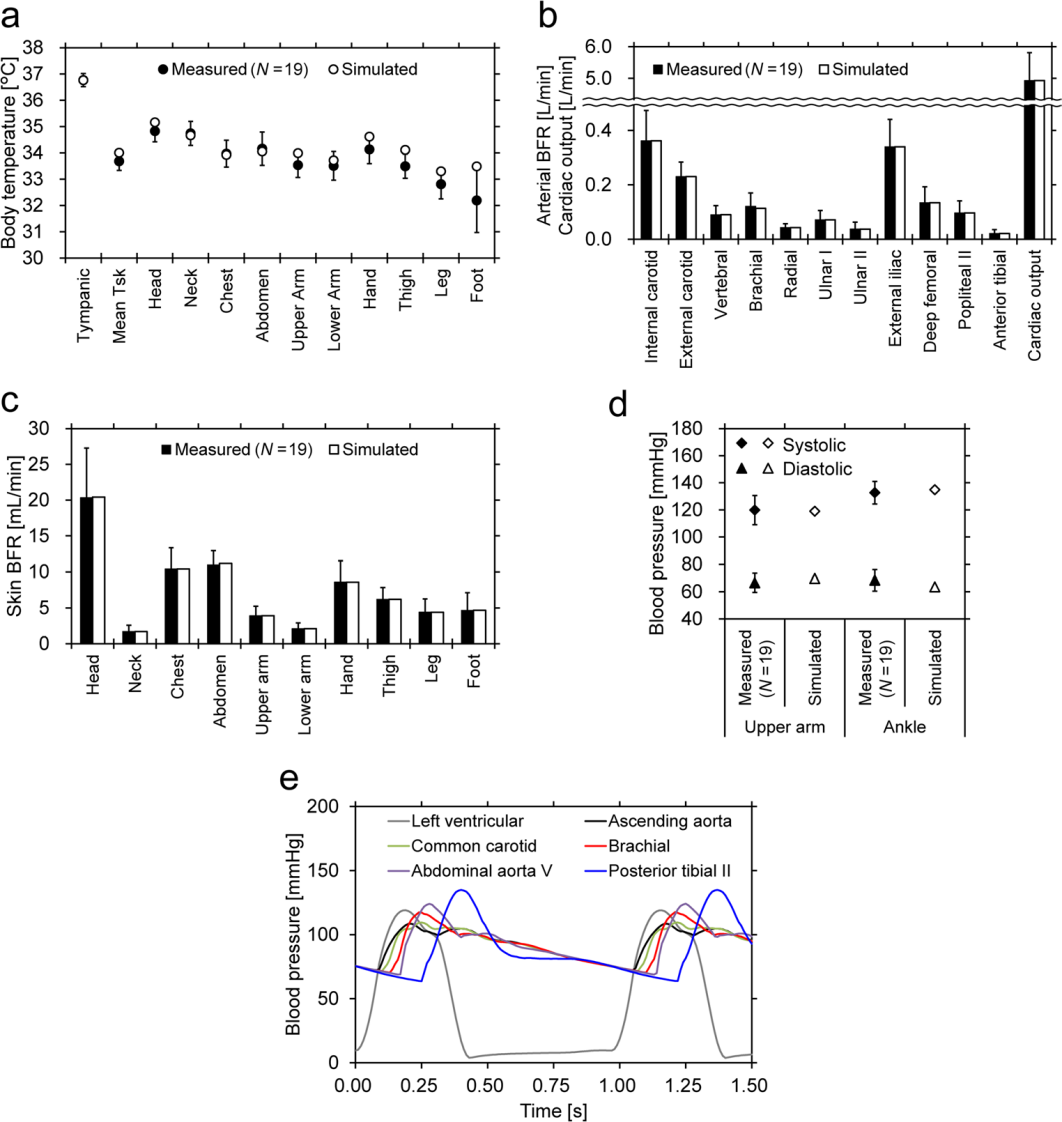
Simulation results and experimental data under the baseline condition (error bar: standard deviation of the measurements) **a**: body temperature; **b**: arterial blood flow rate and cardiac output; **c**: skin blood flow rate; **d**: blood pressure; **e**: time variation of simulated blood pressure (no experimental data for time variation of blood pressure)

A comparison between the simulation results and experimental data is shown in Fig. 4a–d. The arterial and skin BFRs were reproduced by adjusting the 0-D model. The BTs and BPs simulated with these BFR values were in good agreement with the experimental values. Figure 4e shows the time variation of the simulated BP at several sites. The systolic/diastolic BPs at the upper arm and ankle shown in Fig. 4d correspond to the maximum/minimum BPs at the brachial and posterior tibial arteries shown in Fig. 4e, respectively.

## Discussion

The novelty of this study lies in the coupling of a closed-loop cardiovascular model, which handles the entire pathway of an actual circulatory system, with a thermophysiological model. In previous models by Ghaddar and her colleagues (Salloum et al. 2007; Karaki et al. 2013; Rida et al. 2014) and Coccarelli et al. (2016, 2018), the cardiovascular model did not include the venous system. Consequently, these models cannot account for effects such as gravity-induced venous expansion in the lower limbs when people are standing, the accompanying decrease in cardiac filling pressure and stroke volume (SV), or changes in venous volume and cardiac filling pressure caused by alterations in peripheral vascular resistance due to thermally induced vasodilation and vasoconstriction. Therefore, it is not possible to fairly simulate the changes in BP caused by variations in posture or thermal environments. Liu et al. (2023) employed a closed-loop cardiovascular model in their model but excessively simplified the structure of the arterial system. Therefore, it is impossible to accurately simulate the complex pressure wave propagation phenomena, including the reflection of pressure waves from the peripheral ends of the arterial tree. It is also impossible to accurately simulate situations in which thermophysiological responses, baroreflexes, or physical activity cause the BFR to increase or decrease non-uniformly within the body because the circulatory routes were not divided sufficiently. In this study, the CV model represented the arterial system in a form close to its actual structure, and the division of organs and tissues was sufficiently detailed. Therefore, it is possible to simulate BP propagation and various nonuniform blood flow regulation responses in a realistic manner. However, this study did not address the proposal of an autonomic regulation model for reproducing thermoregulatory responses and its coupling with the physical model.

Another advantage of our model is that it is based on actual BFR measurements throughout the body. Table 3 compares the BFR simulated by our model with the basal BFRs of the Stolwijk (1971) and Gordon (1976) models, which are frequently referenced in other conventional thermophysiological models. In our model, the sum of the BFRs in the head and limb segments was based on the measurements obtained using the ultrasound Doppler technique, whereas the BFRs of the segments in the Stolwijk and Gordon models were estimated by adding the BFR per unit mass of each tissue. The BFR of each segment of our model was roughly similar to that of the Gordon model but differed from that of the Stolwijk model. The BFR to the head segment in our model was greater than that of Stolwijk and Gordon, likely because neither Stolwijk nor Gordon considered the BFR to organs in the head other than the brain. Comparing the BFRs of body tissues between our model and Gordon’s, there were apparent differences in the BFRs for skin and fat; however, the sum of the BFRs for skin and fat was roughly similar. Gordon estimated a very low BFR of fat based on the fat metabolic rate. However, past studies (Larsen et al. 1966; Ardilouze et al. 2004) measured abdominal subcutaneous tissue BFR at 26 or 40 mL/min/kg (2.6 or 4.0 mL/min/100 g), although the room temperature and clothing conditions were unspecified. These values are much larger than the fat BFR of the Gordon model, and we believe that our model better represents the actual subcutaneous BFR conditions. Meanwhile, we found a fact when we simulated using our model with replacement of the BFR values from Goto’s to Stolwijk’s and Gordon’s. The fact was that the accuracy of the simulated BT distribution deteriorated with Stolwijk’s BFRs but slightly with Gordon’s BFRs (Fig. S1, Supplementary Material). This suggests that our BFR values are at least more valid than those of Stolwijk.

**Table 3:** Comparison of basal blood flow rates of the body compartments

Segment	Compartment	BFR [mL/min]			BFR per unit mass [mL/min/kg]		
		Goto	Stolwijk	Gordon	Goto	Stolwijk	Gordon
Head	Brain	904.0	750.0	814.0	602.0	419.0	579.0
	Skull	371.0	0.0	0.0	194.0	0.0	0.0
	Muscle	23.2	2.0	10.0	30.0	5.4	31.5
	Fat	46.5	2.2	0.1	79.9	5.9	0.3
	Skin	20.3	24.0	96.2	79.9	88.9	509.0
	Segmental sum	1370.0	778.0	920.0	272.0	194.0	224.0
Neck	Core	4.5	–	0.0	27.7	–	0.0
	Muscle	10.6	–	18.2	30.0	–	31.3
	Fat	4.5	–	0.0	37.1	–	0.3
	Skin	1.7	–	5.6	37.1	–	174.0
	Segmental sum	21.3	–	23.8	31.2	–	25.9
Chest and abdomen	Core	2070.0	3500.0	2830.0	127.0	238.0	155.0
	Muscle	366.0	100.0	377.0	30.0	5.6	31.4
	Fat	75.1	42.7	2.4	16.9	6.0	0.3
	Skin	21.4	35.0	94.9	17.4	25.9	77.4
	Segmental sum	2540.0	3680.0	3300.0	74.1	89.7	81.7
Upper arm and lower arm (right or left)	Core	1.9	7.0	0.0	3.3	6.3	0.0
	Muscle	104.0	9.5	77.5	60.6	5.6	31.4
	Fat	13.8	1.7	0.1	23.0	3.4	0.3
	Skin	6.0	4.2	23.4	23.0	17.4	83.9
	Segmental sum	125.0	22.3	101.0	40.1	6.3	26.2
Hand (right or left)	Core	0.4	0.8	0.0	3.3	6.4	0.0
	Muscle	16.2	2.0	4.4	122.0	57.1	31.4
	Fat	13.0	0.3	0.0	111.0	4.4	0.3
	Skin	8.6	16.7	18.5	111.0	175.0	201.0
	Segmental sum	38.1	19.8	22.9	85.1	59.2	41.9
Thigh and leg (right or left)	Core	4.2	22.4	0.0	3.3	6.4	0.0
	Muscle	254.0	28.6	115.0	51.1	5.6	31.4
	Fat	41.7	4.3	0.4	23.3	3.6	0.3
	Skin	10.6	23.8	35.0	23.2	39.6	71.1
	Segmental sum	311.0	79.1	151.0	36.6	7.6	19.0
Foot (right or left)	Core	1.5	1.3	0.0	3.3	6.2	0.0
	Muscle	12.7	0.2	6.8	51.1	4.8	31.3
	Fat	10.4	0.4	0.1	36.3	3.8	0.3
	Skin	4.7	25.0	15.7	36.3	208.0	123.0
	Segmental sum	29.2	26.9	22.5	26.4	56.1	20.4
Total		4930.0	4750.0	4840.0	74.4	63.8	66.9

Despite its advantages, this study has some limitations. First, the model was developed for an average Japanese male in their 20s, and it cannot predict physiological quantities for individuals with different characteristics, such as size, age, sex, heredity, or pre-existing disorders. To resolve this issue, it is necessary to adjust the model parameter settings to suit individual characteristics, and further studies are required. Second, the distribution of compartment BFRs in each segment was set based on experimental measurements to the extent possible; however, certain assumptions were still necessary. Therefore, there is still room for consideration regarding the validity of these settings. Finally, this study was limited to the development of the physical model that could simulate BT, BP, and BFR only under the baseline condition. To simulate various conditions, an autonomic regulation model must be developed and coupled with the physical model. We plan to report on this issue in our next paper.

## Conclusions

In this study, a new physical model that couples a TNW model for simulating BT with a CV model for simulating BFR and BP was developed. An average Japanese male in their 20s was set as the standard person, and the parameter settings for the person were derived based on recent experimental data and the literature. By applying the parameter settings to the model, it was confirmed that the simulated BT, BFR, and BP values under a thermally neutral and supine condition reproduced the experimental values well.

## Supplementary Information

Below is the link to the electronic supplementary material.

Supplementary Material 1 (DOCX 192 KB)

## Thermal network model

*C*_*I*_: Heat capacity of compartment *I *[J/K]
*C*_*res*_: Sensible heat loss by respiration [W]
*E*_*res*_: Latent heat loss by respiration [W]
*E*_*sk, I*_: Latent heat loss from skin compartment *I* [W]
*M*_*I*_: Metabolic rate of compartment *I* [W]
*q*_*I*,*J*_: Heat gain from node *J* to node*I* [W]
*Q*_*ar*_: Blood flow rate through an artery [m^3^/s]
*Q*_*I*_*,*_*J*_: Blood flow rate from node *J* to node *I *[m^3^/s]
*Q*_*ve*_: Blood flow rate through a vein[m^3^/s]
*t*: Time [s]
*T*_*I*_: Temperature of node *I *[℃]
*TC*_*I*__,__*J*_: Thermal conductance between node *J* to node *I *[W/K]
*ρ**c*: Volumetric specific heat of blood [J/(m^3^･K)]

## Subscripts

*ar*: Artery
*ar_prox*: Proximal artery node of the target artery node
*adj*: Adjacent compartment to artery or vein
*ve*: Vein
*ve_dist*: Distal vein node of the target vein node

## Cardiovascular model

*A*: Cross-sectional area of artery [m^2^]
*A*_0_: Cross-sectional area of artery at reference state (*P* = 0) [m^2^]
*B*: Bernoulli’s resistance of cardiac valve [Pa·s^2^/m^6^]
*C*: Compliance [m^3^/Pa]
*E*: Young’s modulus of vascular wall [Pa]
*E*_0_: Zero-volume elastance of pulmonary circulation [Pa/m^3^]
*E*_*A*_: Active elastance of heart [Pa/m^3^]
*E*_*B*_: Passive elastance of heart [Pa/m^3^]
*e*(*t*): Normalized time-varying elastance of heart [ND]
*g*: Gravitational acceleration [m/s^2^]
*H*: Relative height [m]
*h*: Vascular wall thickness of artery [m]
*L*: Inertial resistance [Pa·s^2^/m^3^]
*l*: Length of artery [m]
*P*: Transmural pressure [Pa]
*Q*: Blood flow rate [m^3^/s]
*R*: Viscosity resistance [Pa·s/m^3^]
*r*: Radius of artery [m]
*r*_0_: Radius of artery at reference state (*P *= 0) [m]
*S*: Viscoelastance of heart or pulmonary circulation [Pa·s/m^3^]
*t*: Time [s]
*T*: Normalized time within a cardiac cycle (= 0 to 1) [ND]
*T*_*cp*_: Normalized duration of atrial or ventricular contraction [ND]
*T*_*cs*_: Normalized time of the beginning of atrial or ventricular contraction [ND]
*T*_*rp*_: Normalized duration of atrial or ventricular relaxation [ND]
*V*: Volume [m^3^]
*x*: Axial coordinate [m]
*δ*: Boundary layer thickness of blood flow in artery (= 0.25*r*) [m]
*ν*: Blood kinematic viscosity [m^2^/s]
*ρ*: Blood density [kg/m^3^]
*σ*: Poisson’s ratio (= 0.5) [ND]
*Φ*: Volume constant of pulmonary circulation [m^3^]

## Subscripts

*dw*: Downstream segment adjacent to *i*
*dist*: Distal end of artery
*i*: Target segment
*prox*: Proximal end of artery
*up*: Upstream segment adjacent to *i*
