# Supplementary material for: Human simulation model for predicting body temperature, blood pressure, and blood flow rate under various thermal exposures — Part 1: development of physical model for average Japanese male in their 20s
Source: Int J Biometeorol. 2026 Feb 3;70(2):48. doi: 10.1007/s00484-025-03072-6 (PMC12868053; doi:10.1007/s00484-025-03072-6)
Supplement: Supplementary file 1 — Supplementary Material 1 (DOCX 192 KB) [file 484_2025_3072_MOESM1_ESM.docx]

# Supplementary Material

**Table S1** Surface area and volume of each segment, and volume of each compartment

| Segment | Surface area  [m^2^]^*2^ | Volume  [10^-3^ m^3^] | Individual volume of compartment [10^-3^ m^3^]^*1^ | | | | |
| --- | --- | --- | --- | --- | --- | --- | --- |
|  |  |  | Brain or Core | Muscle | Fat | Skin | Others |
| Head | 0.1272 | 4.660 | 1.431 | 0.722 | 0.685 | 0.254 | Skull 1.568  *Details:*  *Bone 0.902*  *Viscera 0.528*  *Air 0.139* |
| Neck | 0.0230 | 0.794 | 0.129  *Details:*  *Bone 0.063*  *Viscera 0.066* | 0.330 | 0.144 | 0.046 |  |
| Chest | 0.2407 | 13.016 | 3.305  *Details:*  *Bone 1.492*  *Viscera 0.955*  *Air 0.019*  *Fat 0.893* | 3.860 | 1.667 | 0.481 | R. Heart 0.203  L. Heart 0.203  Lung 3.296 |
| Abdomen | 0.3752 | 21.149 | 9.282  *Details:*  *Bone 2.451*  *Liver 1.150*  *Kidney 0.296*  *Viscera 2.764*  *Air 0.399*  *Fat 2.222* | 7.540 | 3.576 | 0.750 |  |
| Upper arm (right or left) | 0.0850 | 2.067 | 0.246 | 1.069 | 0.513 | 0.170 |  |
| Lower arm (right or left) | 0.0456 | 0.960 | 0.128 | 0.530 | 0.189 | 0.091 |  |
| Hand (right or left) | 0.0389 | 0.420 | 0.081 | 0.123 | 0.138 | 0.078 |  |
| Thigh (right or left) | 0.1309 | 5.436 | 0.419 | 3.228 | 1.469 | 0.262 |  |
| Leg (right or left) | 0.0982 | 2.729 | 0.433 | 1.422 | 0.635 | 0.196 |  |
| Foot (right or left) | 0.0642 | 0.991 | 0.294 | 0.232 | 0.337 | 0.128 |  |
| *1 The volume of each compartment was obtained by classifying 55 tissues and organs in the voxel model by Nagaoka et al. (2004). The muscle, fat, and skin compartments generally correspond to the original tissue classification of the voxel model, whereas for the fat of the chest and abdomen, subcutaneous fat was categorized into the fat compartment and visceral fat into the core compartment.  *2 The surface area was calculated by dividing the skin volume of the segment by 2 mm (the side length of a voxel) because Nagaoka et al. (2004) classified the first surface layer of the voxel model as skin. | | | | | | | |

**Table S2** Volumetric specific heat, density (Freitas 1999), basal metabolic rate (Elia 1992), and thermal conductivity (Freitas 1999) of each tissue or organ

|  | Volumetric specific heat [J/(m^3^·K)] | Density [kg/m^3^] | Basal metabolic rate [W/kg] | Thermal conductivity [W/(m·K)] |
| --- | --- | --- | --- | --- |
| Bone | 2390000 | 1500 | 0.581 | 1.160 |
| Brain | 3860000 | 1050 | 11.600 | 0.528 |
| Heart | 3940000 | 1060 | 21.300 | –^*1^ |
| Lung | 2240000 | 603 | 0.581 | 0.282 |
| Liver | 3780000 | 1050 | 17.4 [W]^*2^ | 0.565 |
| Kidneys | 4080000 | 1050 | 21.300 | 0.544 |
| Viscera | 3890000 | 1050 | 0.581 | 0.544 |
| Muscle | 3640000 | 1070 | 0.630 | 0.372 |
| Fat | 1960000 | 850 | 0.218 | 0.190 |
| Skin | 3770000 | 1000 | 0.581 | 0.335 |
| Blood | 3820000 | 1050 | 0.000 | 0.549 |
| Air | 1150 | 1.14 | 0.000 | 0.026 |
| *1 Heat transfer between the heart and lung via blood flow was considered, but that by conduction was neglected. Therefore, the thermal conductivity of the heart was not considered.  *2 The liver of the voxel model by Nagaoka et al. (2004) was smaller than the typical size, which would cause an underestimation of the liver metabolic rate. Therefore, the basal metabolic rate for the whole liver in Elia (1992) was used. | | | | |

**Table S3** Derivation of thermal conductance between compartments

| Shape | Method |  |
| --- | --- | --- |
| Sphere (head) | The center of gravity radius *rm_I_* is calculated using the following equation: $\frac{4}{3}\pi\left(r_{I}^{3}-rm_{I}^{3}\right)=\frac{1}{2}\times\frac{4}{3}\pi\left(r_{I}^{3}-r_{I-1}^{3}\right)$   The thermal conductance *TC_I_*_,_*_I_*_+1_ is derived as: $TC_{I,I+1}=\frac{1}{\frac{rm_{I}^{-1}-r_{I}^{-1}}{4\pi\lambda_{I}}+\frac{r_{I}^{-1}-rm_{I+1}^{-1}}{4\pi\lambda_{I+1}}}$  |  |
| Cylinder | The center of gravity radius *rm_I_* is calculated using the following equation: $\pi\left(r_{I}^{2}-rm_{I}^{2}\right)=\frac{1}{2}\times\pi\left(r_{I}^{2}-r_{I-1}^{2}\right)$   The thermal conductance *TC_I_*_,_*_I_*_+1_ is derived as: $TC_{I,I+1}=\frac{l}{\frac{\ln\left(r_{I}/rm_{I}\right)}{2\pi\lambda_{I}}+\frac{\ln\left(rm_{I+1}/r_{I}\right)}{2\pi\lambda_{I+1}}}$  |  |
| Cylinder and hemisphere (hand and foot) | The center of gravity radius *rm_I_* is calculated using the following equation: $\begin{matrix} \frac{2}{3}\pi\left(r_{I}^{3}-rm_{I}^{3}\right)+\pi\left(r_{I}^{2}-rm_{I}^{2}\right)l \\ =\frac{1}{2}\left\{\frac{2}{3}\pi\left(r_{I}^{3}-r_{I-1}^{3}\right)+\pi\left(r_{I}^{2}-r_{I-1}^{2}\right)l\right\} \end{matrix}$   The thermal conductance *TC_I_*_,_*_I_*_+1_ is derived as: $TC_{I,I+1}=\frac{1}{\frac{rm_{I}^{-1}-r_{I}^{-1}}{2\pi\lambda_{I}}+\frac{r_{I}^{-1}-rm_{I+1}^{-1}}{2\pi\lambda_{I+1}}}+\frac{l}{\frac{\ln\left(r_{I}/rm_{I}\right)}{2\pi\lambda_{I}}+\frac{\ln\left(rm_{I+1}/r_{I}\right)}{2\pi\lambda_{I+1}}}$  |  |
| *l* Length of segment [m]  *r_I_*, *rm_I_* Radius and center of gravity radius of compartment *I* [m]  *λ_I_* Thermal conductivity of compartment *I* [W/(m·K)] | | |

**Table S4** Derivation of thermal conductance between artery and vein or between artery/vein and adjacent compartment

| Type | Method |
| --- | --- |
| Artery and vein | The thermal conductance between the artery and the vein *TC_ar_*_,_*_ve_* is derived as^*1,2^: $TC_{ar,ve}=\frac{2\pi r_{ar}l\times\alpha}{\frac{1}{H_{ar}}+\frac{h_{ar}}{\lambda_{ar}}+\frac{h_{ve}}{\lambda_{ve}}+\frac{1}{H_{ve}}}\times n_{ar}$   The convective heat transfer coefficient between the blood and the arterial wall *H_ar_* or the venous wall *H_ve_* is derived as^*3^: $H_{ar}=Nu\frac{\lambda_{bl}}{d_{ar}} \left(∵Nu=\frac{H_{ar}d_{ar}}{\lambda_{bl}}\right) , H_{ve}=Nu\frac{\lambda_{bl}}{d_{ve}} \left(∵Nu=\frac{H_{ve}d_{ve}}{\lambda_{bl}}\right)$  |
| Artery/vein and adjacent compartment | The thermal conductance between the artery and the adjacent compartment *TC_ar_*_,_*_I_* is derived as: $TC_{ar,I}=\left[\frac{1}{2\pi r_{ar}H_{ar}l}+\frac{1}{\beta_{ar}}\right]^{-1}\times\left(1-\alpha\right)\times n_{ar}$   The thermal conductance between the vein and the adjacent compartment *TC_ve_*_,_*_I_* is derived as: $TC_{ve,I}=\left[\frac{1}{2\pi r_{ve}H_{ve}l}+\frac{1}{\beta_{ve}}\right]^{-1}\times\frac{2\pi r_{ve}n_{ve}-\alpha\times2\pi r_{ar}n_{ar}}{2\pi r_{ve}n_{ve}}\times n_{ve}$   In cases where the artery and vein are located at the center of the segment (abdomen, upper arm, lower arm, thigh, and leg^*4^): $\beta_{ar}=\frac{2\pi\lambda_{I}l}{\ln\left(rm_{I}/r_{ar}\right)} , \beta_{ve}=\frac{2\pi\lambda_{I}l}{\ln\left(rm_{I}/r_{ve}\right)}$ 　　  In cases where the artery and vein are located between the compartment layers (hand and foot), and when the thermal conductance with the inner compartment layer *I* is calculated: $\beta_{ar}=\frac{2\pi\lambda_{I}l}{\ln\left(r_{I}/rm_{I}\right)}\times\frac{\pi r_{ar}}{2\pi r_{I}} , \beta_{ve}=\frac{2\pi\lambda_{I}l}{\ln\left(r_{I}/rm_{I}\right)}\times\frac{\pi r_{ve}}{2\pi r_{I}}$ 　　  In cases where the artery and vein are located between the compartment layers (hand and foot), and when the thermal conductance with the outer compartment layer *I* is calculated: $\beta_{ar}=\frac{2\pi\lambda_{I}l}{\ln\left(rm_{I}/r_{I-1}\right)}\times\frac{\pi r_{ar}}{2\pi r_{I-1}} , \beta_{ve}=\frac{2\pi\lambda_{I}l}{\ln\left(rm_{I}/r_{I-1}\right)}\times\frac{\pi r_{ve}}{2\pi r_{I-1}}$ 　　  In cases where the artery and vein are located at the center of gravity of the compartment *I* (neck^*5^): $\beta_{ar}=\infty, \beta_{ve}=\infty$ 　　 |
| *d_ar_*, *d_ve_* Diameters of artery and vein [m]  *h_ar_*, *h_ve_* Wall thickness of artery and vein [m]  *l* Length of artery or vein (= length of segment) [m]  *Nu* Nusselt number (= 3.66)  *n_ar_*, *n_ve_* Number of arteries and veins in the segment  *r_ar_*, *r_ve_* Radii of artery and vein [m]  *α* Ratio of artery surface area that exchanges heat with vein(s)  *λ_ar_*, *λ_bl_*, *λ_I_*, *λ_ve_* Thermal conductivity of arterial wall, blood, compartment *I*, and venous wall [W/(m·K)] | |
| *1 The ratio *α* of the arterial surface area that exchanges heat with the veins was assumed to be 0.75 for the upper arms, lower arms, and legs, and 0.25 for the neck, abdomen, thighs, hands, and feet. This was estimated based on the fact that two veins run parallel to one artery in the upper arms, lower arms, and legs, and a network structure of veins connects the two veins and surrounds the artery. A single vein runs parallel to a single artery in the neck, abdomen, thighs, hands, and feet.  *2 The wall thickness of the arteries was set as equivalent to that of the arteries in the CV model, while that of the veins was given half the thickness of the arteries in the same segment, referencing Levick (2010).  *3 The convective heat transfer between blood and the vessel wall was assumed to be the developed laminar flow in a circular tube with a constant inner wall temperature, and a Nusselt number of 3.66 was used to calculate the convective heat transfer coefficient, referencing the Japan Society of Mechanical Engineers (2014).  *4 For the upper arms, lower arms, thighs, and legs, both the artery/vein and the core (i.e., bone) are located within the muscle, and they are positioned somewhat apart from each other. Therefore, for the sake of modeling, it was assumed that both the artery/vein and core were at the center of the muscle.  *5 The artery and vein of the neck were assumed to be located at the center of gravity radius of the muscle. | |

**Table S5** Derived thermal conductance between nodes

| Segment | Node | Thermal conductance with the following node in the same segment [W/K] | | | | | | |
| --- | --- | --- | --- | --- | --- | --- | --- | --- |
|  |  | (A) | (B) | (C) | (D) | (E) | (F) | (G) |
| Head | (A) Brain |  | 1.312 |  |  |  |  |  |
|  | (B) Skull | 1.312 |  | 4.406 |  |  |  |  |
|  | (C) Muscle |  | 4.406 |  | 4.304 |  |  |  |
|  | (D) Fat |  |  | 4.304 |  | 6.558 |  |  |
|  | (E) Skin |  |  |  | 6.558 |  |  |  |
|  | (F) Artery |  |  |  |  |  |  |  |
|  | (G) Vein |  |  |  |  |  |  |  |
| Neck | (A) Core |  | 0.268 |  |  |  |  |  |
|  | (B) Muscle | 0.268 |  | 0.415 |  | 3.362 | 3.549 |  |
|  | (C) Fat |  | 0.415 |  | 1.043 |  |  |  |
|  | (D) Skin |  |  | 1.043 |  |  |  |  |
|  | (E) Artery |  | 3.362 |  |  |  | 0.423 |  |
|  | (F) Vein |  | 3.549 |  |  | 0.423 |  |  |
| Chest | (A) Right heart |  |  |  |  |  |  |  |
|  | (B) Left heart |  |  |  |  |  |  |  |
|  | (C) Lung |  |  |  | 1.502 |  |  |  |
|  | (D) Core |  |  | 1.502 |  | 4.353 |  |  |
|  | (E) Muscle |  |  |  | 4.353 |  | 4.869 |  |
|  | (F) Fat |  |  |  |  | 4.869 |  | 10.629 |
|  | (G) Skin |  |  |  |  |  | 10.629 |  |
| Abdomen | (A) Core |  | 3.220 |  |  | 1.154 | 1.308 |  |
|  | (B) Muscle | 3.220 |  | 5.484 |  |  |  |  |
|  | (C) Fat |  | 5.484 |  | 12.391 |  |  |  |
|  | (D) Skin |  |  | 12.391 |  |  |  |  |
|  | (E) Artery | 1.154 |  |  |  |  | 0.674 |  |
|  | (F) Vein | 1.308 |  |  |  | 0.674 |  |  |
| Upper arm (right or left) | (A) Core |  | 0.976 |  |  |  |  |  |
|  | (B) Muscle | 0.976 |  | 1.543 |  | 0.111 | 0.656 |  |
|  | (C) Fat |  | 1.543 |  |  |  |  | 4.539 |
|  | (D) Skin |  |  |  |  |  |  | 26.649 |
|  | (E) Artery |  | 0.111 |  |  |  | 0.865 |  |
|  | (F) Vein |  | 0.656 |  |  | 0.865 |  |  |
|  | (G) Superficial vein |  |  | 4.539 | 26.649 |  |  |  |
| Lower arm (right or left) | (A) Core |  | 0.612 |  |  |  |  |  |
|  | (B) Muscle | 0.612 |  | 1.058 |  | 0.101 | 0.591 |  |
|  | (C) Fat |  | 1.058 |  |  |  |  | 3.552 |
|  | (D) Skin |  |  |  |  |  |  | 14.154 |
|  | (E) Artery |  | 0.101 |  |  |  | 0.874 |  |
|  | (F) Vein |  | 0.591 |  |  | 0.874 |  |  |
|  | (G) Superficial vein |  |  | 3.552 | 14.154 |  |  |  |
| Hand (right or left) | (A) Core |  | 1.333 |  |  | 0.367 | 0.453 |  |
|  | (B) Muscle | 1.333 |  | 1.398 |  | 0.159 | 0.199 |  |
|  | (C) Fat |  | 1.398 |  |  |  |  | 2.998 |
|  | (D) Skin |  |  |  |  |  |  | 11.165 |
|  | (E) Artery | 0.367 | 0.159 |  |  |  | 0.641 |  |
|  | (F) Vein | 0.453 | 0.199 |  |  | 0.641 |  |  |
|  | (G) Superficial vein |  |  | 2.998 | 11.165 |  |  |  |
| Thigh (right or left) | (A) Core |  | 0.658 |  |  |  |  |  |
|  | (B) Muscle | 0.658 |  | 1.256 |  | 0.333 | 0.372 |  |
|  | (C) Fat |  | 1.256 |  |  |  |  | 3.907 |
|  | (D) Skin |  |  |  |  |  |  | 42.253 |
|  | (E) Artery |  | 0.333 |  |  |  | 0.361 |  |
|  | (F) Vein |  | 0.372 |  |  | 0.361 |  |  |
|  | (G) Superficial vein |  |  | 3.907 | 42.253 |  |  |  |
| Leg (right or left) | (A) Core |  | 1.120 |  |  |  |  |  |
|  | (B) Muscle | 1.120 |  | 1.686 |  | 0.184 | 1.069 |  |
|  | (C) Fat |  | 1.686 |  |  |  |  | 5.005 |
|  | (D) Skin |  |  |  |  |  |  | 31.101 |
|  | (E) Artery |  | 0.184 |  |  |  | 1.746 |  |
|  | (F) Vein |  | 1.069 |  |  | 1.746 |  |  |
|  | (G) Superficial vein |  |  | 5.005 | 31.101 |  |  |  |
| Foot (right or left) | (A) Core |  | 2.173 |  |  | 0.448 | 0.558 |  |
|  | (B) Muscle | 2.173 |  | 1.891 |  | 0.313 | 0.392 |  |
|  | (C) Fat |  | 1.891 |  |  |  |  | 3.595 |
|  | (D) Skin |  |  |  |  |  |  | 19.408 |
|  | (E) Artery | 0.448 | 0.313 |  |  |  | 1.337 |  |
|  | (F) Vein | 0.558 | 0.392 |  |  | 1.337 |  |  |
|  | (G) Superficial vein |  |  | 3.595 | 19.408 |  |  |  |
| Note: The thermal conductivities of body tissues and organs necessary for the calculation of thermal conductance are listed in Table S2. The thermal conductance between the superficial vein and fat and between the superficial vein and skin was calculated by assuming that the superficial vein was a zero-thickness compartment located between the fat and skin. | | | | | | | | |

**Table S6** Values of the 0-D model heart parameters

|  | *E_A_* [Pa/m^3^] | *E_B_* [Pa/m^3^] | *T_cs_* [s/s] | *T_cp_* [s/s] | *T_rp_* [s/s] | *S* [Pa·s/m^3^] | *R* [Pa·s/m^3^] | *B* [Pa·s^2^/m^6^] | *L* [Pa·s^2^/m^3^] |
| --- | --- | --- | --- | --- | --- | --- | --- | --- | --- |
| R. atrium | 8.00E+06 | 9.33E+06 | 0.80 | 0.17 | 0.17 | 500.0×*P*_1_ | 1.33E+05 | 2.13E+09 | 2.67E+04 |
| R. ventricle | 7.33E+07 | 6.67E+06 | 0.00 | 0.30 | 0.15 | 500.0×*P*_2_ | 6.67E+05 | 3.33E+09 | 6.67E+04 |
| L. atrium | 9.33E+06 | 1.20E+07 | 0.80 | 0.17 | 0.17 | 500.0×*P*_6_ | 1.33E+05 | 2.13E+09 | 2.67E+04 |
| L. ventricle | 3.67E+08 | 1.07E+07 | 0.00 | 0.30 | 0.15 | 500.0×*P*_7_ | 1.33E+06 | 3.33E+09 | 6.67E+04 |
| *P*_1_, *P*_2_, *P*_6_, and *P*_7_ are the pressures at the right atrium, right ventricle, left atrium, and left ventricle, respectively. | | | | | | | | | |

**Table S7** Values of the 0-D model pulmonary circulation and vena cava parameters

|  | *E*_0_ [Pa/m^3^] | *Φ* [m^3^] | *C* [m^3^/Pa] | *R* [Pa·s/m^3^] | *L* [Pa·s^2^/m^3^] | *S* [Pa·s/m^3^] |
| --- | --- | --- | --- | --- | --- | --- |
| Pulmonary arteries | 2.67E+06 | 2.00E-05 | – | 5.33E+06 | 6.67E+04 | 1.33E+06 |
| Pulmonary capillaries | 2.67E+06 | 6.00E-05 | – | 5.33E+06 | 6.67E+04 | 1.33E+06 |
| Pulmonary veins | 2.67E+06 | 2.00E-04 | – | 6.67E+05 | 6.67E+04 | 1.33E+06 |
| Superior vena cava | – | – | 3.75E-08 | 6.67E+04 | 6.67E+04 | – |
| Inferior vena cava | – | – | 5.63E-08 | 6.67E+04 | 6.67E+04 | – |
| Abdominal vena cava | – | – | 5.63E-08 | 6.67E+04 | 6.67E+04 | – |

**Table S8** Blood flow rates of the body compartments

| Segment | BFR of the compartment [mL/min] (BFR per unit mass of the compartment [mL/min/kg]) | | | | | | | | | |
| --- | --- | --- | --- | --- | --- | --- | --- | --- | --- | --- |
|  | Brain or Core^*1^ | | Muscle^*2^ | | Fat^*3^ | | Skin^*4^ | | Skull^*5^ | |
| Head | 904.0 | (602.0) | 23.2 | (30.0) | 46.5 | (79.9) | 20.3 | (79.9) | 371.0 | (194.0) |
| Neck | 4.5 | (27.7) | 10.6 | (30.0) | 4.5 | (37.1) | 1.7 | (37.1) |  | |
| Chest | 97.1 | (24.6) | 124.0 | (30.0) | 30.6 | (21.6) | 10.4 | (21.6) |  | |
| Abdomen | 1980.0 | (198.0) | 242.0 | (30.0) | 44.5 | (14.6) | 11.0 | (14.6) |  | |
| Upper arm (R. or L.) | 1.2 | (3.3) | 34.3 | (30.0) | 10.0 | (23.0) | 3.9 | (23.0) |  | |
| Lower arm (R. or L.) | 0.6 | (3.3) | 69.5 | (122.0) | 3.7 | (23.1) | 2.1 | (23.1) |  | |
| Hand (R. or L.) | 0.4 | (3.3) | 16.2 | (122.0) | 13.0 | (111.0) | 8.6 | (111.0) |  | |
| Thigh (R. or L.) | 2.1 | (3.3) | 176.0 | (51.1) | 29.6 | (23.7) | 6.2 | (23.7) |  | |
| Leg (R. or L.) | 2.1 | (3.3) | 77.7 | (51.1) | 12.1 | (22.4) | 4.4 | (22.4) |  | |
| Foot (R. or L.) | 1.5 | (3.3) | 12.7 | (51.1) | 10.4 | (36.3) | 4.7 | (36.3) |  | |
| *1 The brain BFR was set to match the measurements by Goto et al. (2024). The core BFR of the neck and chest was set such that the BFR per metabolic rate was equivalent to that of the muscle. The core BFR of the abdomen was set as the cardiac output (CO) minus the BFRs of all other segments and compartments. The core BFR of the limbs (i.e., bone BFR) was set with reference to Freitas (1999).  *2 The muscle BFR of the lower arm and hand was calculated by subtracting the BFR of non-muscle compartments from the total BFR of the lower arm and hand, as estimated by Goto et al. (2024), and the remaining BFR was distributed so that the muscle BFR per unit mass was identical for both the lower arm and hand. The muscle BFR of the thigh, leg, and foot was calculated by subtracting the BFR of non-muscle compartments from the total BFR of the thigh, leg, and foot, as estimated by Goto et al. (2024), and the remaining BFR was distributed so that the muscle BFR per unit mass was identical for all the thigh, leg, and foot. The muscle BFR of the other segments was set with reference to Freitas (1999).  *3 The fat (subcutaneous tissue) BFR was assumed to be the same BFR per unit mass as the skin, because the subcutaneous BFR per unit mass measured in past studies (Larsen et al. 1966; Ardilouze 2004) was much closer to the skin BFR per unit mass measured by Goto et al. (2024) than that estimated from the fat metabolic rate.  *4 The skin BFR was set to match the measurements by Goto et al. (2024).  *5 The skull BFR was set as the total BFR of the head estimated by Goto et al. (2024) minus the BFRs of all compartments other than the skull. | | | | | | | | | | |

**Table S9** Values of the 0-D model peripheral circulation parameters under the baseline condition
(*C* [m^3^/Pa], *R* [Pa·s/m^3^], and *L* [Pa·s^2^/m^3^])

| ID No. | Distal end of artery | Arteriole | | | Capillary | | | Venule | | | Vein | | |
| --- | --- | --- | --- | --- | --- | --- | --- | --- | --- | --- | --- | --- | --- |
|  | *R*_0_ | *C*_1_ | *R*_2-5_ | *L*_2-5_ | *C*_6-9_ | *R*_6-9_ | *L*_6-9_ | *C*_10-13_ | *R*_10-13_ | *L*_10-13_ | *C*_14_ | *R*_14_ | *L*_14_ |
|  |  |  | (2 to 5 from the top) | | (6 to 9 from the top) | | | (10 to 13 from the top) | | |  |  |  |
| 4, 21 | 6.9E+07 | 3.1E-10 | 1.7E+09 | 1.4E+06 | 3.1E-11 | 8.3E+07 | 2.3E+05 | 9.3E-10 | 2.7E+07 | 4.1E+05 | 1.1E-08 | 6.6E+06 | 4.6E+05 |
|  |  |  | – | – | – | – | – | – | – | – |  |  |  |
|  |  |  | – | – | – | – | – | – | – | – |  |  |  |
|  |  |  | – | – | – | – | – | – | – | – |  |  |  |
| 5, 22 | 1.1E+08 | 2.0E-10 | 3.2E+09 | 2.1E+06 | 1.6E-11 | 1.6E+08 | 3.5E+05 | 4.9E-10 | 5.1E+07 | 6.3E+05 | 7.2E-09 | 1.0E+07 | 5.7E+05 |
|  |  |  | 5.1E+10 | 3.4E+07 | 1.0E-12 | 2.5E+09 | 5.6E+06 | 3.1E-11 | 8.1E+08 | 1.0E+07 |  |  |  |
|  |  |  | 2.5E+10 | 1.7E+07 | 2.0E-12 | 1.3E+09 | 2.8E+06 | 6.1E-11 | 4.0E+08 | 5.0E+06 |  |  |  |
|  |  |  | 5.8E+10 | 3.9E+07 | 9.0E-13 | 2.9E+09 | 6.4E+06 | 2.7E-11 | 9.2E+08 | 1.1E+07 |  |  |  |
| 7, 25 | 2.8E+08 | 7.7E-11 | 6.8E+09 | 2.8E+06 | 7.7E-12 | 3.4E+08 | 4.6E+05 | 2.3E-10 | 1.1E+08 | 8.2E+05 | 1.9E-09 | 2.7E+07 | 9.2E+05 |
|  |  |  | – | – | – | – | – | – | – | – |  |  |  |
|  |  |  | – | – | – | – | – | – | – | – |  |  |  |
|  |  |  | – | – | – | – | – | – | – | – |  |  |  |
| 9, 27 | 2.4E+09 | 9.2E-12 | 2.7E+11 | 3.8E+07 | 1.9E-13 | 1.3E+10 | 6.3E+06 | 5.8E-12 | 4.3E+09 | 1.1E+07 | 1.0E-09 | 2.2E+08 | 2.7E+06 |
|  |  |  | 1.1E+11 | 1.6E+07 | 4.5E-13 | 5.7E+09 | 2.7E+06 | 1.4E-11 | 1.8E+09 | 4.8E+06 |  |  |  |
|  |  |  | 2.7E+11 | 3.8E+07 | 1.9E-13 | 1.3E+10 | 6.3E+06 | 5.8E-12 | 4.3E+09 | 1.1E+07 |  |  |  |
|  |  |  | 7.1E+11 | 1.0E+08 | 7.3E-14 | 3.5E+10 | 1.7E+07 | 2.2E-12 | 1.1E+10 | 3.0E+07 |  |  |  |
| 11, 29 | 4.9E+08 | 4.5E-11 | 4.8E+11 | 1.5E+08 | 1.1E-13 | 2.4E+10 | 2.5E+07 | 3.3E-12 | 7.6E+09 | 4.4E+07 | 4.8E-09 | 4.6E+07 | 1.2E+06 |
|  |  |  | 1.7E+10 | 5.3E+06 | 3.1E-12 | 8.4E+08 | 8.8E+05 | 9.2E-11 | 2.7E+08 | 1.6E+06 |  |  |  |
|  |  |  | 5.8E+10 | 1.8E+07 | 9.0E-13 | 2.9E+09 | 3.0E+06 | 2.7E-11 | 9.2E+08 | 5.4E+06 |  |  |  |
|  |  |  | 1.5E+11 | 4.6E+07 | 3.5E-13 | 7.4E+09 | 7.7E+06 | 1.0E-11 | 2.4E+09 | 1.4E+07 |  |  |  |
| 13, 31 | 5.8E+08 | 3.8E-11 | 3.2E+12 | 9.1E+08 | 1.6E-14 | 1.6E+11 | 1.5E+08 | 4.9E-13 | 5.1E+10 | 2.7E+08 | 9.4E-10 | 5.5E+07 | 1.3E+06 |
|  |  |  | 2.9E+10 | 8.3E+06 | 1.8E-12 | 1.4E+09 | 1.4E+06 | 5.4E-11 | 4.6E+08 | 2.5E+06 |  |  |  |
|  |  |  | 5.4E+11 | 1.6E+08 | 9.6E-14 | 2.7E+10 | 2.6E+07 | 2.9E-12 | 8.6E+09 | 4.6E+07 |  |  |  |
|  |  |  | 9.6E+11 | 2.7E+08 | 5.4E-14 | 4.8E+10 | 4.5E+07 | 1.6E-12 | 1.5E+10 | 8.1E+07 |  |  |  |
| 14, 32 | – | – | 2.8E+12 | 7.9E+08 | 1.9E-14 | 1.4E+11 | 1.3E+08 | 5.6E-13 | 4.4E+10 | 2.4E+08 | – | – | – |
|  |  |  | 6.9E+10 | 2.0E+07 | 7.6E-13 | 3.4E+09 | 3.3E+06 | 2.3E-11 | 1.1E+09 | 5.8E+06 |  |  |  |
|  |  |  | 8.6E+10 | 2.4E+07 | 6.1E-13 | 4.3E+09 | 4.1E+06 | 1.8E-11 | 1.4E+09 | 7.3E+06 |  |  |  |
|  |  |  | 1.3E+11 | 3.7E+07 | 4.0E-13 | 6.4E+09 | 6.1E+06 | 1.2E-11 | 2.1E+09 | 1.1E+07 |  |  |  |
| 16, 34 | 7.1E+08 | 3.1E-11 | 2.0E+12 | 5.3E+08 | 2.5E-14 | 1.0E+11 | 8.8E+07 | 7.6E-13 | 3.3E+10 | 1.6E+08 | 6.8E-10 | 6.7E+07 | 1.5E+06 |
|  |  |  | 1.9E+10 | 4.8E+06 | 2.8E-12 | 9.2E+08 | 8.0E+05 | 8.4E-11 | 3.0E+08 | 1.4E+06 |  |  |  |
|  |  |  | 3.5E+11 | 9.0E+07 | 1.5E-13 | 1.7E+10 | 1.5E+07 | 4.5E-12 | 5.5E+09 | 2.7E+07 |  |  |  |
|  |  |  | 6.1E+11 | 1.6E+08 | 8.5E-14 | 3.0E+10 | 2.6E+07 | 2.5E-12 | 9.8E+09 | 4.7E+07 |  |  |  |
| 17, 35 | 6.8E+08 | 3.2E-11 | 3.8E+12 | 9.9E+08 | 1.4E-14 | 1.9E+11 | 1.6E+08 | 4.2E-13 | 6.0E+10 | 2.9E+08 | 8.0E-10 | 6.4E+07 | 1.4E+06 |
|  |  |  | 3.4E+10 | 9.0E+06 | 1.5E-12 | 1.7E+09 | 1.5E+06 | 4.6E-11 | 5.4E+08 | 2.7E+06 |  |  |  |
|  |  |  | 6.4E+11 | 1.7E+08 | 8.2E-14 | 3.2E+10 | 2.8E+07 | 2.4E-12 | 1.0E+10 | 5.0E+07 |  |  |  |
|  |  |  | 1.1E+12 | 3.0E+08 | 4.6E-14 | 5.6E+10 | 4.9E+07 | 1.4E-12 | 1.8E+10 | 8.8E+07 |  |  |  |
| 18, 36 | – | – | 3.3E+12 | 8.6E+08 | 1.6E-14 | 1.6E+11 | 1.4E+08 | 4.8E-13 | 5.2E+10 | 2.6E+08 | – | – | – |
|  |  |  | 8.1E+10 | 2.1E+07 | 6.5E-13 | 4.0E+09 | 3.5E+06 | 1.9E-11 | 1.3E+09 | 6.3E+06 |  |  |  |
|  |  |  | 1.0E+11 | 2.7E+07 | 5.2E-13 | 5.0E+09 | 4.4E+06 | 1.6E-11 | 1.6E+09 | 7.9E+06 |  |  |  |
|  |  |  | 1.5E+11 | 4.0E+07 | 3.4E-13 | 7.5E+09 | 6.6E+06 | 1.0E-11 | 2.4E+09 | 1.2E+07 |  |  |  |
| 38 | 9.7E+07 | 2.2E-10 | 6.3E+09 | 4.4E+06 | 8.2E-12 | 3.1E+08 | 7.3E+05 | 2.5E-10 | 1.0E+08 | 1.3E+06 | 2.9E-09 | 9.2E+06 | 5.4E+05 |
|  |  |  | 5.0E+09 | 3.5E+06 | 1.0E-11 | 2.5E+08 | 5.7E+05 | 3.1E-10 | 7.9E+07 | 1.0E+06 |  |  |  |
|  |  |  | 2.0E+10 | 1.4E+07 | 2.6E-12 | 1.0E+09 | 2.3E+06 | 7.8E-11 | 3.2E+08 | 4.2E+06 |  |  |  |
|  |  |  | 5.9E+10 | 4.1E+07 | 8.8E-13 | 2.9E+09 | 6.8E+06 | 2.6E-11 | 9.4E+08 | 1.2E+07 |  |  |  |
| 40 | 1.6E+08 | 1.4E-10 | – | – | – | – | – | – | – | – | 2.4E-09 | 1.5E+07 | 7.0E+05 |
|  |  |  | 5.1E+09 | 2.7E+06 | 1.0E-11 | 2.5E+08 | 4.6E+05 | 3.1E-10 | 8.1E+07 | 8.1E+05 |  |  |  |
|  |  |  | 2.1E+10 | 1.1E+07 | 2.5E-12 | 1.0E+09 | 1.9E+06 | 7.5E-11 | 3.3E+08 | 3.3E+06 |  |  |  |
|  |  |  | 8.4E+10 | 4.5E+07 | 6.2E-13 | 4.2E+09 | 7.5E+06 | 1.9E-11 | 1.3E+09 | 1.3E+07 |  |  |  |
| 43 | 1.8E+08 | 1.2E-10 | 4.4E+09 | 2.2E+06 | 1.2E-11 | 2.2E+08 | 3.7E+05 | 3.6E-10 | 6.9E+07 | 6.6E+05 | 2.2E-07 | 1.7E+07 | 7.4E+05 |
|  |  |  | – | – | – | – | – | – | – | – |  |  |  |
|  |  |  | – | – | – | – | – | – | – | – |  |  |  |
|  |  |  | – | – | – | – | – | – | – | – |  |  |  |
| 45 | 2.7E+08 | 8.1E-11 | 6.5E+09 | 2.7E+06 | 8.0E-12 | 3.2E+08 | 4.5E+05 | 2.4E-10 | 1.0E+08 | 8.1E+05 | 8.0E-09 | 2.6E+07 | 9.0E+05 |
|  |  |  | – | – | – | – | – | – | – | – |  |  |  |
|  |  |  | – | – | – | – | – | – | – | – |  |  |  |
|  |  |  | – | – | – | – | – | – | – | – |  |  |  |
| 46 | 1.2E+08 | 1.9E-10 | 2.8E+09 | 1.8E+06 | 1.9E-11 | 1.4E+08 | 3.0E+05 | 5.6E-10 | 4.4E+07 | 5.3E+05 | 1.6E-08 | 1.1E+07 | 5.9E+05 |
|  |  |  | – | – | – | – | – | – | – | – |  |  |  |
|  |  |  | – | – | – | – | – | – | – | – |  |  |  |
|  |  |  | – | – | – | – | – | – | – | – |  |  |  |
| 48 | 4.6E+07 | 4.7E-10 | 1.1E+09 | 1.1E+06 | 4.7E-11 | 5.6E+07 | 1.9E+05 | 1.4E-09 | 1.8E+07 | 3.3E+05 | 4.0E-08 | 4.4E+06 | 3.7E+05 |
|  |  |  | – | – | – | – | – | – | – | – |  |  |  |
|  |  |  | – | – | – | – | – | – | – | – |  |  |  |
|  |  |  | – | – | – | – | – | – | – | – |  |  |  |
| 50, 52 | 5.6E+07 | 3.9E-10 | 1.4E+09 | 1.2E+06 | 3.8E-11 | 6.7E+07 | 2.1E+05 | 1.1E-09 | 2.2E+07 | 3.7E+05 | 4.0E-08 | 5.4E+06 | 4.1E+05 |
|  |  |  | – | – | – | – | – | – | – | – |  |  |  |
|  |  |  | – | – | – | – | – | – | – | – |  |  |  |
|  |  |  | – | – | – | – | – | – | – | – |  |  |  |
| 54 | 3.4E+08 | 6.3E-11 | 8.3E+09 | 3.1E+06 | 6.3E-12 | 4.1E+08 | 5.1E+05 | 1.9E-10 | 1.3E+08 | 9.1E+05 | 1.6E-08 | 3.3E+07 | 1.0E+06 |
|  |  |  | – | – | – | – | – | – | – | – |  |  |  |
|  |  |  | – | – | – | – | – | – | – | – |  |  |  |
|  |  |  | – | – | – | – | – | – | – | – |  |  |  |
| 57, 72 | 3.6E+08 | 6.0E-11 | – | – | – | – | – | – | – | – | 9.2E-09 | 3.5E+07 | 1.0E+06 |
|  |  |  | 1.0E+10 | 3.6E+06 | 5.1E-12 | 5.0E+08 | 6.0E+05 | 1.5E-10 | 1.6E+08 | 1.1E+06 |  |  |  |
|  |  |  | 8.2E+10 | 3.0E+07 | 6.3E-13 | 4.1E+09 | 4.9E+06 | 1.9E-11 | 1.3E+09 | 8.8E+06 |  |  |  |
|  |  |  | 3.3E+11 | 1.2E+08 | 1.6E-13 | 1.7E+10 | 2.0E+07 | 4.7E-12 | 5.3E+09 | 3.6E+07 |  |  |  |
| 59, 74 | 1.8E+08 | 1.2E-10 | 4.6E+11 | 2.3E+08 | 1.1E-13 | 2.3E+10 | 3.9E+07 | 3.4E-12 | 7.3E+09 | 6.9E+07 | 8.8E-09 | 1.8E+07 | 7.4E+05 |
|  |  |  | 5.4E+09 | 2.7E+06 | 9.6E-12 | 2.7E+08 | 4.5E+05 | 2.9E-10 | 8.6E+07 | 8.1E+05 |  |  |  |
|  |  |  | 3.2E+10 | 1.6E+07 | 1.6E-12 | 1.6E+09 | 2.7E+06 | 4.8E-11 | 5.1E+08 | 4.8E+06 |  |  |  |
|  |  |  | 1.5E+11 | 7.8E+07 | 3.4E-13 | 7.6E+09 | 1.3E+07 | 1.0E-11 | 2.4E+09 | 2.3E+07 |  |  |  |
| 61, 76 | 3.0E+08 | 7.2E-11 | 7.6E+11 | 3.0E+08 | 6.9E-14 | 3.8E+10 | 5.0E+07 | 2.1E-12 | 1.2E+10 | 8.9E+07 | 7.7E-09 | 2.9E+07 | 9.5E+05 |
|  |  |  | 8.9E+09 | 3.5E+06 | 5.9E-12 | 4.4E+08 | 5.8E+05 | 1.8E-10 | 1.4E+08 | 1.0E+06 |  |  |  |
|  |  |  | 5.3E+10 | 2.1E+07 | 9.8E-13 | 2.6E+09 | 3.5E+06 | 3.0E-11 | 8.4E+08 | 6.2E+06 |  |  |  |
|  |  |  | 2.5E+11 | 9.9E+07 | 2.1E-13 | 1.3E+10 | 1.7E+07 | 6.2E-12 | 4.0E+09 | 3.0E+07 |  |  |  |
| 63, 78 | 8.5E+08 | 2.6E-11 | 9.2E+11 | 2.2E+08 | 5.7E-14 | 4.6E+10 | 3.6E+07 | 1.7E-12 | 1.5E+10 | 6.4E+07 | 2.8E-09 | 8.0E+07 | 1.6E+06 |
|  |  |  | 2.5E+10 | 6.0E+06 | 2.1E-12 | 1.3E+09 | 9.9E+05 | 6.2E-11 | 4.0E+08 | 1.8E+06 |  |  |  |
|  |  |  | 1.6E+11 | 3.8E+07 | 3.2E-13 | 8.1E+09 | 6.4E+06 | 9.6E-12 | 2.6E+09 | 1.1E+07 |  |  |  |
|  |  |  | 4.5E+11 | 1.1E+08 | 1.2E-13 | 2.2E+10 | 1.7E+07 | 3.5E-12 | 7.1E+09 | 3.1E+07 |  |  |  |
| 65, 80 | 1.2E+09 | 1.9E-11 | 2.5E+12 | 5.0E+08 | 2.1E-14 | 1.2E+11 | 8.3E+07 | 6.3E-13 | 4.0E+10 | 1.5E+08 | 7.1E-10 | 1.1E+08 | 1.9E+06 |
|  |  |  | 6.9E+10 | 1.4E+07 | 7.6E-13 | 3.4E+09 | 2.3E+06 | 2.3E-11 | 1.1E+09 | 4.1E+06 |  |  |  |
|  |  |  | 4.4E+11 | 8.9E+07 | 1.2E-13 | 2.2E+10 | 1.5E+07 | 3.5E-12 | 7.0E+09 | 2.6E+07 |  |  |  |
|  |  |  | 1.2E+12 | 2.4E+08 | 4.3E-14 | 6.0E+10 | 4.1E+07 | 1.3E-12 | 1.9E+10 | 7.3E+07 |  |  |  |
| 66, 81 | – | – | 1.1E+12 | 2.3E+08 | 4.6E-14 | 5.6E+10 | 3.8E+07 | 1.4E-12 | 1.8E+10 | 6.8E+07 | – | – | – |
|  |  |  | 1.3E+11 | 2.6E+07 | 4.0E-13 | 6.5E+09 | 4.3E+06 | 1.2E-11 | 2.1E+09 | 7.8E+06 |  |  |  |
|  |  |  | 1.6E+11 | 3.2E+07 | 3.3E-13 | 7.9E+09 | 5.3E+06 | 9.9E-12 | 2.5E+09 | 9.5E+06 |  |  |  |
|  |  |  | 3.5E+11 | 7.1E+07 | 1.5E-13 | 1.8E+10 | 1.2E+07 | 4.4E-12 | 5.6E+09 | 2.1E+07 |  |  |  |
| 68, 83 | 6.1E+08 | 3.6E-11 | 6.6E+11 | 1.8E+08 | 7.9E-14 | 3.3E+10 | 3.0E+07 | 2.4E-12 | 1.1E+10 | 5.5E+07 | 3.9E-09 | 5.8E+07 | 1.4E+06 |
|  |  |  | 1.8E+10 | 5.1E+06 | 2.9E-12 | 9.1E+08 | 8.4E+05 | 8.6E-11 | 2.9E+08 | 1.5E+06 |  |  |  |
|  |  |  | 1.2E+11 | 3.3E+07 | 4.4E-13 | 5.8E+09 | 5.4E+06 | 1.3E-11 | 1.9E+09 | 9.7E+06 |  |  |  |
|  |  |  | 3.2E+11 | 8.9E+07 | 1.6E-13 | 1.6E+10 | 1.5E+07 | 4.9E-12 | 5.1E+09 | 2.7E+07 |  |  |  |
| 69, 84 | 6.4E+08 | 3.4E-11 | 1.4E+12 | 3.7E+08 | 3.8E-14 | 6.9E+10 | 6.2E+07 | 1.1E-12 | 2.2E+10 | 1.1E+08 | 4.3E-10 | 6.1E+07 | 1.4E+06 |
|  |  |  | 3.8E+10 | 1.0E+07 | 1.4E-12 | 1.9E+09 | 1.7E+06 | 4.1E-11 | 6.1E+08 | 3.1E+06 |  |  |  |
|  |  |  | 2.4E+11 | 6.6E+07 | 2.1E-13 | 1.2E+10 | 1.1E+07 | 6.4E-12 | 3.9E+09 | 2.0E+07 |  |  |  |
|  |  |  | 6.7E+11 | 1.8E+08 | 7.8E-14 | 3.3E+10 | 3.0E+07 | 2.3E-12 | 1.1E+10 | 5.4E+07 |  |  |  |
| 70, 85 | – | – | 6.2E+11 | 1.7E+08 | 8.3E-14 | 3.1E+10 | 2.8E+07 | 2.5E-12 | 9.9E+09 | 5.0E+07 | – | – | – |
|  |  |  | 7.2E+10 | 1.9E+07 | 7.2E-13 | 3.6E+09 | 3.2E+06 | 2.2E-11 | 1.1E+09 | 5.8E+06 |  |  |  |
|  |  |  | 8.7E+10 | 2.4E+07 | 6.0E-13 | 4.3E+09 | 3.9E+06 | 1.8E-11 | 1.4E+09 | 7.0E+06 |  |  |  |
|  |  |  | 1.9E+11 | 5.3E+07 | 2.7E-13 | 9.7E+09 | 8.8E+06 | 8.0E-12 | 3.1E+09 | 1.6E+07 |  |  |  |
| Note: The peripheral circulations of Nos. 14, 18, 32, 36, 66, 70, 81, and 85 share the *R*_0_, *C*_1_, *C*_14_, *R*_14_, and *L*_14_ with those of Nos. 13, 17, 31, 35, 65, 69, 80, and 84, respectively. | | | | | | | | | | | | | |

**Table S10** Boundary conditions for simulating the baseline condition

|  | Head | Neck | Chest | Abdomen | Upper arm  (R. or L.) | Lower arm  (R. or L.) | Hand  (R. or L.) | Thigh  (R. or L.) | Leg  (R. or L.) | Foot  (R. or L.) |
| --- | --- | --- | --- | --- | --- | --- | --- | --- | --- | --- |
| Total heat transfer coefficient *h_t_*  [W/(m^2^･K)]^*1^ | 8.12 | 8.12 | 7.56 | 7.86 | 6.72 | 10.69 | 9.16 | 8.50 | 9.46 | 8.21 |
| Clothing insulation *I_cl_*  [m^2^･K/W]^*1,2^ | 0.08 | 0.08 | 0.03 | 0.08 | 0.02 | 0.05 | 0.05 | 0.09 | 0.03 | 0.02 |
| Convective heat transfer coefficient *h_c_*  [W/(m^2^･K)]^*3^ | 3.82 | 3.82 | 3.86 | 3.96 | 2.72 | 6.79 | 5.46 | 4.30 | 4.76 | 2.81 |
| Permeation efficiency of clothing *i_cl_*  [ND]^*4^ | 0.90 | 0.90 | 0.90 | 0.45 | 0.90 | 0.90 | 0.90 | 0.90 | 0.90 | 0.90 |
| *1 We measured using a thermal manikin.  *2 The clothing insulation consisted of underpants, short pants, beach bed, and hair wig on a thermal manikin.  *3 The convective heat transfer coefficient was obtained as the measured total heat transfer coefficient minus the radiant heat transfer coefficient in the literature (Ichihara et al. 1996).  *4 The typical value for normal clothing (Gagge et al. 1986) was applied for the abdomen covered by underpants and short pants. For the other segments lying on a highly permeable beach bed made of polyester netting, the values were assumed to be twice the typical value. Evaporative heat loss from the skin *E_sk_* [W] was calculated using the following formula: $E_{sk}=\frac{wL_{a}A_{sk}}{1/h_{c}+I_{cl}/i_{cl}}\left(p_{s,sk}-p_{a}\right)$   *A_sk_* Skin surface area [m^2^]  *L_a_* Lewis number [K/Pa]  *p_a_* Ambient vapor pressure [Pa]  *p_s_*_,_*_sk_* Saturated vapor pressure at skin [Pa]  *w* Skin wettedness (= 0.06) [ND] | | | | | | | | | | |

**Fig. S1** Body temperatures simulated using Goto model with different blood flow rates

**a:** comparison of simulated body temperatures before and after replacing the BFR values with Stolwijk’s

**b:** comparison of simulated body temperatures before and after replacing the BFR values with Gordon’s

**References cited in the Supplementary Material**

Ardilouze JL, Fielding BA, Currie JM, Frayn KN, Karpe F (2004) Nitric oxide and β-adrenergic stimulation are major regulators of preprandial and postprandial subcutaneous adipose tissue blood flow in humans. Circulation 109(1): 47-52. <https://doi.org/10.1161/01.CIR.0000105681.70455.73>

Elia M (1992) Organ and tissue contribution to metabolic rate. In: Kinney JM and Tucker HN (eds) Energy Metabolism: Tissue Determinants and Cellular Corollaries. Raven Press, New York, 61-79.

Freitas RA (1999) Nanomedicine, Volume I: Basic Capabilities. Landes Bioscience, Austin, Texas.

Gagge AP, Fobelets AP, Berglund LG (1986) A standard predictive index of human response to the thermal environment. ASHRAE Transactions 92(2B): 709-731.

Goto T, Niu Z, Chiba Y, Amano K, Saijo Y (2024) Human body temperature and cardiovascular response to changes in ambient temperature and body posture. Building and Environment 266: 112085. <https://doi.org/10.1016/j.buildenv.2024.112085>

Ichihara M, Saitou M, Nishimura M, Tanabe S (1996) Measurement of convective and radiative heat transfer coefficients of standing and sitting human body by using a thermal manikin. Journal of Architecture and Planning (Transactions of AIJ) 62(501): 45-51. <https://doi.org/10.3130/aija.62.45_5>

Japan Society of Mechanical Engineers (2014) JSME Mechanical Engineers' Handbook, α5: Thermal Engineering. Maruzen, Tokyo.

Larsen OA, Lassen NA, Quaade F (1966) Blood flow through human adipose tissue determined with radioactive Xenon. Acta Physiologica Scandinavica 66(3): 337-345. <https://doi.org/10.1111/j.1748-1716.1966.tb03208.x>

Levick JR (2010) Cardiovascular Physiology. Fifth edition. Hodder Arnold, London.

Nagaoka T, Watanabe S, Sakurai K, Kunieda E, Watanabe S, Taki M, Yamanaka Y (2004) Development of realistic high-resolution whole-body voxel models of Japanese adult males and females of average height and weight, and application of models to radio-frequency electromagnetic-field dosimetry. Physics in Medicine & Biology 49: 1-15. <https://doi.org/10.1088/0031-9155/49/1/001>
